# Effects of behavioural interventions for preventing obesity in young children from ethnic minority backgrounds: a systematic review of randomised controlled trials

**DOI:** 10.1186/s13690-026-01951-x

**Published:** 2026-05-18

**Authors:** Erica J. Cook, Jane Williams, Louisa Donald, Ffion Curtis, Chris Bridle, Amy Ridge, Jamie M. Whitehall, Faye Powell

**Affiliations:** 1 School of Sports, Psychology and Social Sciences, University of Bedfordshire, Luton, UK; 2 School of Psychology, University of Birmingham, Dubai, United Arab Emirates; 3 Liverpool Reviews and Implementation Group (LRiG), University of Liverpool, Liverpool, UK; 4 President’s Office, University of Kurdistan Hewlêr, Kurdistan, Iraq; 5 Institute for Health and Wellbeing Research, University of Bedfordshire, Luton, UK

**Keywords:** Systematic review, Obesity prevention, Children, Ethnic minorities, Behaviour change

## Abstract

**Background:**

Tackling childhood obesity represents a significant public health challenge associated with several adverse health and psychological outcomes, with increased prevalence in ethnically diverse populations. Early intervention is required to promote healthy weight from early childhood and reduce health inequalities. A systematic review was conducted to synthesise evidence from randomised trials of behavioural interventions that target obesity prevention among young ethnic minority children aged 0–5 years in high-income countries.

**Methods:**

Medline, PsycINFO, Scopus and PubMed were searched for articles published between 1995 and 2023 to examine the theoretical and cultural adaptation strategies associated with the most effective interventions. A narrative synthesis using SWiM guidelines was conducted to summarise the data.

**Results:**

Forty-four articles reporting on thirty-eight unique RCT intervention studies are reported. Around one-third of the interventions resulted in a difference in weight, which favoured the intervention when compared to the control. Behavioural interventions that utilise deep and tailored cultural adaptation strategies may be more effective in changing dietary practices and weight outcomes but may be less important in increasing physical activity levels. The theoretical domains and behaviour change techniques associated with the most promising success are reported.

**Conclusions:**

Collaborative partnerships with diverse families alongside wider community stakeholders may offer a more effective solution to ensure obesity prevention interventions remain tailored to the local context and provide sustainable solutions that can deliver increased impact.

**Supplementary Information:**

The online version contains supplementary material available at 10.1186/s13690-026-01951-x.

**Table:** 

Text box 1. **Contributions to the literature**	
• This review offers new insights into cultural adaptation strategies and the behaviour change techniques used to promote healthy weight in children from diverse communities.	
• This review provides an increased understanding of the theoretical mechanisms and cultural adaptation that may influence intervention success.	
• Obesity prevention interventions delivered across multiple settings may be most effective when targeting children's weight-related behaviours.	
• The findings reinforce the importance of engaging with communities to co-create relevant, inclusive, culturally tailored obesity prevention strategies to improve intervention effectiveness.	

## Introduction

Childhood obesity represents one of the most significant public health challenges of the 21st century. Currently, in the UK, one in four children in their first year of school are classified obese, rising to one in three by the age of 11 [1]. Whilst the prevalence of childhood obesity has been associated with high-income Western countries such as the UK and USA, the gap is now closing with higher prevalence being found in middle- and low-income countries [2]. More recent evidence suggests that if current trends continue, it is forecasted that by 2050, one-third of children and adolescents globally will have overweight or obesity [3]. The prevalence of childhood obesity is not equally distributed across the UK population, with minority ethnic children, particularly those from black and South Asian communities, being more likely to be overweight or obese [4–6]. Childhood obesity is also strongly associated with deprivation; children who live in a deprived area are twice as likely to be obese when compared to their more affluent counterparts [7]. However, whilst deprivation is important, it has failed to explain the pattern of ethnic disparities in childhood obesity [8]. Given these trends, and in line with the UK’s levelling-up strategy [9], early intervention to positively impact behaviours that contribute to obesity and weight is vitally important, with increasing evidence that early prevention can promote healthy weight from early childhood into adulthood and reduce existing health inequalities [10].

Behavioural interventions have been widely used to prevent and reduce childhood obesity alongside improving children’s health-related quality of life, educational attainment, lifetime achievement, and self-esteem [11]. Most interventions have focused on improving breastfeeding rates and supporting the transition to solid foods, alongside interventions that promote healthy eating and physical activity [12]. Systematic reviews have examined the effectiveness of such early behavioural interventions for preventing childhood obesity in 0-5-year-old children [10, 13, 14] and the impact of these across different contexts, e.g. community-based [15, 16], home and family-based [17–20] and more recently across preschool settings [21]. However, although it is widely accepted that theoretically informed behavioural interventions may be more likely to be successful [22–24], there is limited evidence synthesis of the behavioural interventions that target young children, particularly those from ethnic minority populations [22–24]. There is limited evidence to date which has determined which, if any, of these behaviour change techniques (BCTs) [25] are most likely to successfully promote positive behaviour change in high-risk populations, such as those from ethnic minority communities, where complex and interrelating contextual and cultural factors influence childhood obesity [26].

In addition, emerging evidence has suggested that interventions to prevent childhood obesity across minority ethnic populations that are culturally adapted are more relevant, feasible, and effective [27]. However, the approaches and strategies used to ‘culturally adapt’ an intervention can vary in breadth, depth, and rigour [27, 28]. Interventions may utilise peripheral-level strategies such as surface structure adaptations (e.g., visual aids) or higher-level engagement strategies such as ‘constituent involving’ (e.g., participatory community approaches) to adapt interventions [27, 29]. Likewise, interventions may be culturally adapted for a broader population or specific sub-population/s or even at an individual level, which may additionally impact relevance and efficacy [29, 30]. Therefore, a current understanding of what, if any, cultural adaptations make an intervention more effective in minority ethnic populations is vitally important.

Previous systematic reviews have explored behavioural interventions to prevent overweight and obesity in young children, with a more recent focus on discovering which behaviour change techniques might be aligned with the most success [24]. However, there is a lack of evidence which has examined the theoretical and contextual factors that may influence intervention effects in minority ethnic children [21]. To develop and implement interventions that can successfully prevent childhood obesity in high-risk, ethnically diverse population groups, it is essential to understand better which intervention characteristics are associated with the most success. This systematic review aimed to (a) examine the effects of behavioural interventions to prevent childhood obesity in young (aged 0–5 years) ethnic minority children and (b) examine what behaviour change techniques and cultural adaptation strategies are associated with the most effective interventions.

## Materials and methods

A protocol for this systematic review was registered in advance and is available in PROSPERO (CRD42022348116) [31]. This review has been conducted and reported according to the PRISMA 2020 statement [32] (Supplementary File S1) and Synthesis without meta-analysis (SWiM) guidelines [33].

### Inclusion criteria

- Pre-school children (and carers/parents of) with a mean age of five years or less from a minority ethnic background relative to the country of study (including refugees, asylum seekers or migrants).
- To be included, minority ethnic populations had to represent fifty per cent or more of the total sample and have either low socioeconomic status, low-income, or low education (high school or below), and/or be recruited from a low-income area /setting.
- Randomised Controlled Trials (RCTs) with any comparison included within the primary studies (e.g., wait-list control, treatment as usual, attentive control) that report on the primary and/or secondary outcomes of interest identified as the most important modifiable predictors of weight in young children [34].

### Search strategy

A search strategy co-developed with the research team and an academic health sciences librarian included a combination of terms from medical subject headings (MeSH) and keywords based on the predefined PICO components: (1) ethnicity, (2) child, (3) lifestyle modification/intervention, (4) feeding or physical activity.

Electronic databases, namely, PubMed, Medline, PsycINFO, Cumulative Index to Nursing and Allied Health Literature (CINAHL), Scopus and the Cochrane Central Register of Controlled Trials (CENTRAL), were searched for relevant published studies. Obesity prevention trials in young children (under eight years of age) were not conducted before 1995 [35, 36]; therefore, we restricted the search to only include randomised controlled trials published on or after 1 January 1995 up to the 1st November 2023. See Supplementary File 2 for each of the search strategies used. Database searching was supplemented with Google Scholar and reviewing global key nutrition agency databases (e.g., World Food Programme Harvest Plus) for relevant intervention evaluations. Reference lists of included studies and relevant systematic reviews were reviewed.

### Study selection

**Fig. 1:**
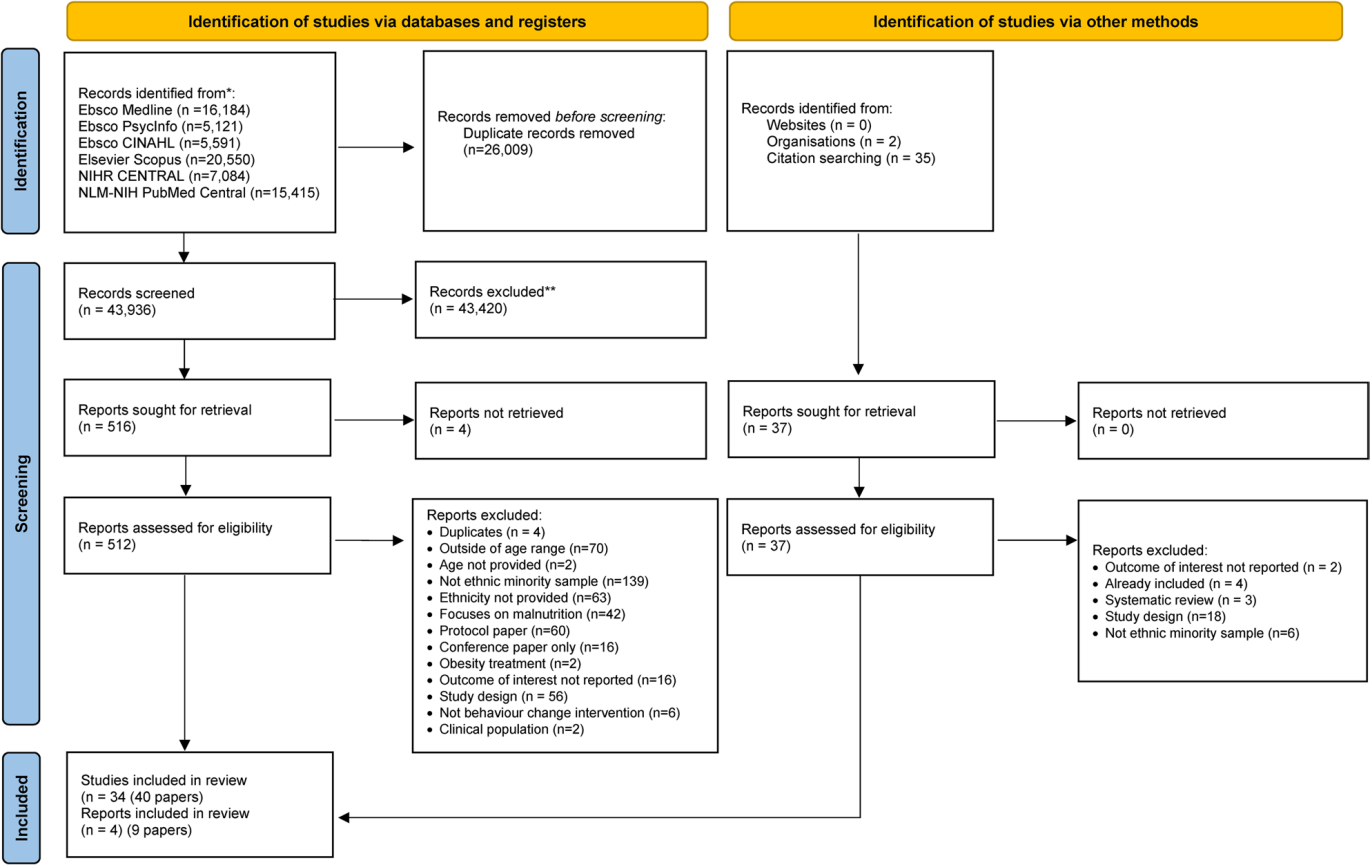
PRISMA flow diagram for study inclusion

Retrieved references were downloaded and imported into the EndNote X9 reference manager system [37], where duplicates were removed. The references were then imported into Rayyan software [38], which was used to manage the screening process. The titles and abstracts were independently blind and double screened using the predefined inclusion criteria (reviewers E.J.C, J.W, F.P, and L.D.) and any discrepancies were resolved through discussion. Full texts were obtained and independently double-screened (reviewers E.J.C., J.W, F.P, and L.D.) for references meeting the eligibility criteria, with reasons for exclusion recorded (see Fig. 1).

### Data extraction

Data from all included studies were extracted by one reviewer (L.D.) into Excel using a data extraction template (see Table 1) that was pre-piloted on a sub-sample of studies (*n* = 2) before use to ensure consistency. All extractions were verified for accuracy by a second reviewer (J.Wh.).

**Table 1:** Data extraction table

Extraction categories	Extraction items
General	Author(s); data of extraction, country where study is located; conflicts of interest.
Study Characteristics	Design: aims/objectives of the study; study design; inclusion and exclusion criteria; recruitment and sampling methods (including unit of randomisation and blinding); unit of allocation; power calculations.
Participant characteristics	Population type; inclusion and exclusion criteria; number of participants (total and by condition); age; gender; ethnicity; religion; socio-economic status, income level, clinical differences (inc. weight status) and any differences at baseline and attrition/drop-out.
Intervention features	Description of the intervention(s); intervention duration; theoretical basis; intervention setting; control condition(s); intervention provider; delivery format; materials; procedures; behaviour change techniques; TIDieR guidelines [114]; cultural adaptations/tailoring.
Outcomes	Primary outcomes: unit of measurement; type of measurement (e.g., subjective); follow-up duration and frequency; mean and standard deviation at baseline, post intervention, and follow-up; effectiveness at postintervention and follow-up for any objective anthropometric measures (e.g., BMI, Z scores, waist to hip ratio etc.); effect size.
	Secondary outcomes: adverse effects; effectiveness at post intervention and follow-up for any of the following child or parental related outcomes. • *Child behavioural outcomes*: physical activity; dietary intake (energy and micro/macronutrient); eating behaviour; sedentary activity; and media-related activity. • Parental related outcomes: dietary-related parenting practices (e.g., breastfeeding, time of introduction of solids, feeding styles) and attitudes; physical-related parenting practices and attitudes; media-related parenting practices and attitudes.

### Quality assessment

Two reviewers (E.J.C, F.P.) independently assessed the methodological quality of the studies using the Critical Appraisal Skills Programme (CASP) tool for randomised trials [39]. The CASP checklist provides a list of screening questions that assess whether the basic study design is valid for a randomised controlled trial, whether the study was methodologically sound, what the results were, and whether the results will help locally. Studies were graded based on how many of the assessment requirements were met: low (< 50%), medium (50%–79%), and high (≤ 80%).

### Behaviour change techniques

Two experienced reviewers (J.W., J.Wh.) identified and coded the BCTs of papers for both experimental and control conditions according to the CALO-RE taxonomy [25]. BCTs were coded for both experimental and control groups following the technique described by Prestwich et al. (2014), where BCTs were coded as + 1 if the BCT was used in the experimental condition only; 0 if used in the experimental and control condition or neither; and 1 if the BCT was used in the control group only [40]. Once agreed, the review authors mapped the specific BCTs to the Capability Opportunity Motivation - Behaviour (COM-B) [41] model using the Theoretical Domains Framework (TDF) [42]. Another reviewer verified all codes, and no discrepancies were noted. In line with previous research, a percentage effectiveness ‘ratio’ was then used to assess the effectiveness of the BCTs used and the TDF domains targeted [24]. This was calculated by dividing the number of times a component of an intervention featured within an effective RCT by the number of times it was a component of all trials, including non-effective trials [24].

### Cultural adaptation strategies

A weighted, point-based scoring system determined the depth and breadth of cultural adaptation [27] used within each intervention study. Five categories of cultural adaption [29] were assessed: peripheral strategies, which ensure the audio or visual elements of an intervention reflect the lived experiences of the ethnic group (e.g. music, pictures, foods, clothing, locations, and people); evidential strategies, which address scientific evidence and target health issues relevant to the intended audience, constituent-involving strategies, which incorporate input from the target community, socio-cultural strategies, which imbed social and cultural values into the intervention design and linguistic strategies, which develop culturally equivalent translations of materials and measurement instruments.

Each of these categories were assigned a weighted base score of 1–4 points [27], with less weight (1 point) assigned to surface structure adaptations (peripheral and evidential strategies) and more weight (2 points) assigned to deep structure adaptations, which have higher levels of cultural sensitivity and more significant community input (constituent-involving and socio-cultural) [28]. Linguistic strategies were weighted highest, with 2 points for translation of materials and 2 points for translation of instruments. A higher weighting was given to interventions tailored to individuals, with less to a sub-group and the least to the ethnic group as a whole. All scores were summed to produce a total score with a normalised percentage obtained by dividing the total adaptation score by 15, yielding the maximum possible score [27]. Three interval ranks, as suggested, were selected, which included minimal adaptation (< 50%), moderate adaptation (≥ 50% and < 75%), and comprehensive adaptation (≥ 75%). All scorings were conducted by one author (F.P.) and were verified by a second author (E.J.C.).

### Outcome measures

The primary outcome of interest was the change in body composition identified through an anthropometric measure (s). Arthrometric measures provide a valuable assessment of nutritional status in children and can identify growth patterns to assess the risk of obesity [43]. Anthropometric outcome measure (s) included changes in Body Mass Index (BMI) and other weight-adjusted height measures, including z-scores, changes in body weight and changes in fat distribution (measured by hip circumference, waist circumference, waist-to-hip ratio, and skinfolds thickness/ratio).

Secondary outcomes included child behavioural outcomes; dietary intake (energy and micro/macronutrient), eating behaviour, physical activity and sedentary and media-related activity, alongside parent-related outcomes; parental feeding practices in infancy (e.g. breastfeeding, weaning) and early childhood) and physical activity and media related parenting practices and attitudes. These outcomes were chosen as they represent modifiable predictors of weight and future weight trajectory in young children [34].

### Narrative synthesis

Heterogeneity precluded the pooled statistical analysis of outcomes (e.g., meta-analysis). The findings from the included studies were synthesised narratively and reported in accordance with the SWiM guidelines [44]. Study and intervention characteristics were summarised and presented as text and Tables (see Tables 2 and 3). Standardised mean differences were used to calculate odds ratios, alongside P values, to determine intervention effects across primary and secondary outcomes. Direction of effect plots [45] for all studies were created for all outcomes to summarise the findings. To facilitate interpretation, key characteristics, including the level of cultural adaptations (minimal, moderate, comprehensive), risk of bias (low, moderate, high), sample size (< 100, ≥ 100), study setting, and population targeted, were included. Harvest plots [46] were also used to visually display intervention effects on the primary outcome (excluding infant feeding) and physical activity. Studies were grouped according to outcomes, study setting and population targeted and then whether the direction of change favoured the intervention compared to the control was noted (as determined by odds ratios) with a visual representation of the level of cultural adaptation and risk of bias, study setting (where relevant) and if the findings were significant to facilitate further interpretation.

**Table 2:** Characteristics of included studies

Author, Year Country	Aim	Target group	Sample characteristics	Study Design (*N*)	Age at start (M/SD)	Follow up	Outcomes
1. Toussaint et al., 2021 Netherlands	To examine the effects of a preschool based intervention for teachers in promoting healthy eating and physical activity in young children.	Teachers based in urban preschools located in a deprived setting	Ethnicity: Dutch int − 18%; con- 20%; Moroccan int − 37%; con − 33%; Turkish int − 16%; con − 20%; Western other int − 7%; con − 11%; non-Western other int − 22%; con − 17% Gender: Children (% female): int – 51%; con – 46	Cluster RCT 41 Preschools (INT *n* = 21; CON *n* = 20) 115 Teachers (INT: *n* = 56, CON: *n* = 59) 249 Children (INT: *n* = 137, CON: *n* = 112); TOTAL = 364	Children: 2.5–3.5 yrs Mean (yrs) Int − 3.0 (0.2); Con − 3.0 (0.2)	Baseline 4 months 9 months	Primary outcome/s Teachers’ knowledge, attitudes, practices, and confidence Secondary outcome/s teachers/child BMI/BMIz, teachers body composition, dietary intake and physical activity
2. Gross et al. 2016; Messito, et al. 2020; Messito et al. 2020 USA	To examine the effects of an intervention on child weight outcomes compared with controls receiving standard care	Pregnant women who were Latina or Hispanic, English-or Spanish-speaking	Ethnicity: Hispanic/Latino int − 100%; con − 100% Gender: Mothers (% female): 100; Children (% female): int – 50; con – 52	RCT INT: *n* = 266; CON: *n* = 267; TOTAL: *N* = 533	Mothers: 32 weeks’ gestational age Mean (yrs) Int − 28.6 (6.1); Con − 27.9 (5.7)	Baseline 1 year 2 years 3 years	Primary outcome/s Child BMIz, obesity prevalence /reduction of weight (weight-for-age z-score)
3. Natale et al., 2014 USA	To assess the effectiveness of a multifaceted obesity prevention intervention on BMI and dietary and physical activity patterns of children	Inner-city multi-ethnic preschool children	Ethnicity: Hispanic int – 65.6%; con – 50.7%; African American int – 19.3%; con – 30.4%; Haitian int – 1.3%; con − 7.5%; Other/unknown int – 13.8%; con – 11.7% Gender: Children (% female): int – 52; con – 51	Cluster RCT 8 childcare centres INT: *n* = 238; CON: *n* = 69; TOTAL: *N* = 307	Children aged 2–5 yrs Age (%) 2 yrs Int − 14.3%; Con – 29; 3 yrs Int − 35.7%; Con − 33.3%; 4 yrs Int − 36.6%); Con − 31.9; 5 yrs Int − 13.5; Con − 5.8%	Baseline 3 months 6 months 9 months 12 months	Primary outcome/s Child BMIz Secondary outcome/s Child dietary intake (junk food, fruit & veg, juice, milk) & physical activity
4. Vaughn et al. (2020) USA	To evaluate the impact of an intervention on children’s diet quality and physical activity	Parents with children aged 3–4 years who were able to write and speak English	Ethnicity: White int − 43.2%; con − 49.9%; black African American int − 38.4%; con − 33.9%; other int − 18.4%; con − 16.2%; Latino int − 11.2%; con − 5.7% Gender: Children (% female): 100	Cluster RCT INT: *n* = 446; CON: *n* = 407; TOTAL: *N* = 853	Children aged 3–4 yrs Mean age (months) Int − 48.27 (6.99); Con − 47.55 (6.92)	Baseline 8–10 months	Primary outcome/s Child diet quality, non-sedentary activity (mins) Secondary outcome/s Child BMIz, Caregivers’ nutrition & physical activity practices
5. Tomyako et al. (2016) USA	To test the efficacy of an obesity prevention toolkit delivered either via (Group 1) home mentors or (Group 2) monthly mailings, on child and adult weight status, nutrition & physical activity and self-efficacy	Children aged 2–5 years free of any major physical or behavioural disorders	Ethnicity: American Indian (group 1) − 91%; (group 2) -92.8%; white/other (group 1) − 9%; (group 2) -7.2% Gender: Children (% female): int – 51; con – 43	RCT Group 1 (home mentors) (*n* = 83); Group 2 (home mailings) (*n* = 67); TOTAL: *N* = 150	Children aged 2–5 yrs Mean (yrs) Int − 4.0 (0.9); Con − 4.0 (0.9)	Baseline 1 year 2 years	Primary outcome/s Child/Adult BMIz /BMI Secondary outcome/s Child /parent dietary intake, screen time, daily physical activity & sedentary time (%), self-efficacy for health-related behaviours, perceived health status
6. Wasser et al. (2020) USA	To explore the effectiveness of a home-based, responsive feeding and care, intervention delivered by trained peer educators to pregnant women and their partners	Mothers aged 18–39 years < 28 weeks’ gestation expecting a single pregnancy	Ethnicity: Non-Hispanic black 100% Gender: Mothers (% female): 100	RCT INT: *n* = 215; CON: *n* = 214; TOTAL: *N* = 429	Pregnant women aged 18–39 yrs Mean (yrs) Int − 26.2 (5.5); Con − 25.3 (5.2)	Baseline Birth 3 months 6 months 9 months 12 months 15 months	Primary outcome/s Child weight-for-length Z-score
7. Annesi et al. (2013) USA	To explore the effectiveness of a cognitive-behavioural treatment on physical activity in African American children	African American children aged 3–5 years	Ethnicity: African American 100% Gender: Children (% female): int – 53; con – 56	Cluster RCT INT: *n* = 154; CON: *n* = 121; TOTAL: *N* = 275	Children aged 3–5 yrs Mean (yrs) Int – 4.6 (0.6); Con – 4.7 (0.3)	Baseline 8 weeks	Primary outcome/s Child BMIz, moderate-to-vigorous physical activity, sedentary time, vigorous physical activity
8. Barkin et al. (2012) USA	To test the effect of a culturally tailored, family-centered, short-term behavioral intervention on BMI in Latino-American preschool children	Latino parents with a child aged 2–6 years old not enrolled in any other healthy lifestyle programmes	Ethnicity: Latino 100% Gender: Children (% female): int – 46; con – 55; Parents (% female): int – 94; con – 95	RCT INT: *n* = 54; CON: *n* = 52; TOTAL: *N* = 106	Children aged 2–6 yrs Mean (yrs) Int – 4.1 (0.9); Con – 4.2 (0.9)	Baseline 3 months	Primary outcome/s Child BMIz
9. Barkin et al. (2018); Heerman et al. (2020) USA	To test the effect of a multicomponent behavioral intervention on child BMI growth trajectories over 36 months among preschool-age children at risk for obesity	Children aged 3–5 years who had a weight that was normal-to-overweight but not yet obese, and who spoke Spanish or English	Ethnicity: Hispanic Mexican int – 61.7%; con – 66.5%; Hispanic non-Mexican int − 30.4%; con − 24.3%; non-Hispanic black int − 6.3%; con − 5.6%; Other int – 1.7%; con – 3.6% Gender: Children (% female): int – 51; con – 53; Parents (% female): int – 99; con – 98	RCT INT: *n* = 304 dyads; CON: *n* = 306 dyads; TOTAL: *N* = 610 dyads	Children aged 3–5 yrs Mean (yrs) Int – 4.3 (0.9); Con – 4.3 (0.9)	Baseline 3 months 9 months 1 year 2 years 3 years	Primary outcome/s Child BMI trajectory (over 3 years) Secondary outcome/s Parent-reported child dietary intake, physical activity & use of community center
10. Beck et al. (2017) USA	To evaluate an educational module for Latino parents about the health effects of sweet beverages	Latino parents with children aged 6 mths to 5 years	Ethnicity: Latino 100% Gender: Children (% female): int – 50; con – 45	RCT INT: *n* = 41; CON: *n* = 41; TOTAL: *N* = 82	Children aged 6 mths to 5 yrs Mean age not reported	Baseline 2 weeks 2 months 3 months	Primary outcome/s Child consumption of sugar-sweetened beverages
11. Black et al. (2021) USA	To reduce the rate of BMI growth of toddlers, through a responsive parenting and maternal lifestyle intervention	Mothers WIC eligible, English speaking with children aged 12–32 months (term birth of > 37 weeks, birthweight > 2,500 g) with no health restrictions	Mom-TOPS/Tot-TOPS/CON Ethnicity: African American Mom-TOPS – 68%; Tot-TOPS – 73%; con – 68%; non-Hispanic white Mom-TOPS − 22%; Tot – TOPS − 21%; con − 24%; Other Mom-TOPS − 10%; Tot TOPS − 6%; con − 8% Gender: Children (% female): Mom-TOPS – 51; Tot-TOPS – 40; con – 49; Parents (% female): 100	RCT INT (Mom-TOPS): *n* = 94; INT (Tot-TOPS): *n* = 92; CON: *n* = 91; TOTAL: *N* = 277	Children aged 12–32 months Mean (mths) Mom-TOPS – 19.98 (5.55); Tot-TOPS – 20.22 (5.56); Con – 20.14 (5.46)	Baseline 6 months 1 year	Primary outcome/s Child BMIz Secondary outcome/s maternal–toddler physical activity, mealtime interactions, diet quality
12. Bürgi et al. (2012) Switzerland	To examine whether a lifestyle intervention on adiposity and fitness was equally effective in children of migrant and/or low education level parents	Migrant preschool aged children who had at least one parent born outside of Switzerland with a low educational level	Ethnicity: Former Yugoslavian 25%; Portuguese 17%; European other 31%; African 12%; Other 15% Gender: Children (% female): 16	Cluster RCT 40 public preschools (*N* = 652)	Children aged 5 yrs Mean (yrs) 5.2 (0.6)	Baseline 1 year	Primary outcome/s Child BMI, aerobic fitness Secondary outcome/s Adiposity (body fat %, waist circumference), motor agility
13. Davis et al. (2016); Cruz et al. (2016) USA	To design, implement, and test the efficacy of a preschool-based, trans community obesity prevention intervention on physical activity at home	Head start Centres with ≥ 2 classrooms with ≥ 15 3-year-old Hispanic or American Indian children	Ethnicity: Hispanic int − 62.6%; con − 56.5%; white int − 55.7%; con − 54.4%; American Indian int − 36.7%; con − 38.2%; Other int − 7.4%; con − 7.3% Gender: Children (% female): int – 51; con – 49	Cluster RCT INT: *n* = 321; CON: *n* = 327; TOTAL: *N* = 648	Children aged 3–5 yrs Mean (yrs) Int – 4.1 (0.7); Con – 4.1 (0.7)	Baseline (Fall 2008) T2 (Spring 2009) T3 (Fall 2009) T4 (Spring 2010)	Primary outcome/s Child BMIz, Secondary outcome/s physical activity
14. Fernandez-Jimenez et al. (2019) USA	To assess the impact of a preschool-based health promotion educational intervention in an underserved community	Pre-schools with children aged 3–5 years	Ethnicity: Non-Hispanic black int − 32.2%; con − 50%; Hispanic/Latino int − 58.8%; con − 42.1%; Other int − 9.1%; con − 7.9% Gender: Children (% female): int – 48; con – 52	Cluster RCT INT: *n* = 398; CON: *n* = 164; TOTAL: *N* = 562	Children aged 3–5 yrs Mean age (yrs) Int – 4.1 (0.6); Con – 4.0 (0.6)	Baseline 5 months	Primary outcome/s Knowledge, attitudes and habits (KAH) scores for diet and physical activity Secondary outcome/s Child BMIz Emotion comprehension score
15. Fiks et al. (2017) USA	To test a Facebook peer-group intervention for low-income mothers to foster behaviors promoting healthy infant growth	Mothers attending obstetrics clinics who were 20–32 weeks pregnant with a BMI > 25	Ethnicity: Hispanic/Latino int − 5%; con − 0%; black African American int − 84%; con − 93%; white int − 7%; con − 5%; other int − 7%; con − 7% Gender: Parents (% female):100	RCT INT: *n* = 43; CON: *n* = 42; TOTAL: *N* = 85	Pregnant women aged 18 yrs or older Mean (yrs) Int − 25.8 (5.2); Con − 27.3 (5.6)	Baseline Birth 2 months 4 months 6 months 9 months	Primary outcome/s Intervention acceptability, maternal & infant feeding practices Secondary outcome/s Child weight-for-length z-score
16. Fisher et al. (2019) USA	To evaluate the efficacy of the 12-week parenting intervention for reducing children’s consumption of “empty” calories	Mothers of children aged 3–5 years who attend the Women, Infants and Child (WIC) clinic	Ethnicity: Black African American int − 86.4%; con − 95%; other int − 13.6%; con − 5% Gender: Parents (% female):100; Children: (% female): int – 61; con – 48	RCT INT: *n* = 59; CON: *n* = 60; TOTAL: *N* = 119	Mothers with children aged 3–5 yr Maternal mean age (yrs) Int − 29.5 (7.3); Con − 30.0 (6.9) Child mean age (yrs) Int – 3.6 (0.1); Con − 3.8 (0.1)	Baseline 12 weeks	Primary outcome/s Child daily energy intake from solid and added sugars Secondary outcome/s child / maternal BMIz/ BMI food parenting practices
17. Fitzgibbon et al. 2002; Stolley et al., 2003; Fitzgibbon et al., 2005[57–59] Phase I USA	To evaluate a multicomponent obesity prevention intervention among diverse, low-income preschoolers	Children aged 3–5 years in a participating classroom and provides consent.	Ethnicity: black int – 99%; con – 80.9%; Latino int- 0% con – 12.7%; Other int − 1.0%; con − 6.6%. Gender: Children: (% female): int – 49.7; con – 50.8% Ethnicity: black int – 15.8%; con – 6.5%; Latino int – 73.3%; con – 89.4%; Mixed int – 10.9%; con – 4.0%. Gender**: **Children: (% female): int – 47.5; con – 51.3%	Cluster RCT 12 Head Start Schools 1999–2001 INT *n* = 197; CON: *n* = 212; TOTAL *N* = 409 12 Head Start Schools 2001–2003 INT: *n* = 202; CON: *n* = 199; TOTAL: *N* = 401	Children aged 3–5 yrs Mean (mths) Int – 48.6 (7.6); Con – 50.8 (6.4) Mean (mths) Int – 50.8 (7.3); Con – 51.0 (7.0)	Baseline (1999) Post intervention 1 Year (2000) 2 Years (2001) Baseline (2001) Post intervention 1 year (2002) 2 years (2003)	Primary outcome/s Child BMIz Secondary outcome/s Child dietary intake, physical activity & screen time
18. Fitzgibbon et al. 2011[61] Kong et al. 2016[57, 62] Phase II USA	To evaluate a multicomponent obesity prevention intervention among diverse, low-income preschoolers	Children aged 3–5 years in a participating classroom and provides consent.	Ethnicity: black int – 97%; con – 91%; Latino int – 1%; con – 5%; Mixed int – 2.0%; con – 4.0%. Gender: Children: (% female): int – 52; con – 55	Cluster RCT 18 schools INT: *n* = 376; CON: *n* = 353; TOTAL: *N* = 729	Children aged 3–5 yrs Mean (mths) Int – 50.7 (6.8); Con – 51.9 (6.3)	Baseline Post intervention 1 year follow up	Primary outcome/s Child BMIz Secondary outcome/s Child dietary intake, physical activity & screen time
19. Fitzgibbon et al., 2013[57, 63] Phase III USA	To test the feasibility of Family-Based Hip-Hop to Health, a school-based obesity prevention intervention for 3–5-year-old Latino children and their parents.	Parent-child dyads were eligible if they were enrolled in participating classroom and provided consent/baseline measurements.	Ethnicity: black int – 0%; con – 4%; Latino int – 96%; con – 92%; Mixed int – 4%; con – 4.0%. Gender**: **Children: (% female): int – 53; con – 50	Cluster RCT 4 schools INT: *n* = 78; CON: *n* = 79; TOTAL: *N* = 157	Children aged 3–5 yrs Mean (mths) Int – 53.7 (4.9); Con – 54.7 (5.1)	Baseline Post intervention 1 year follow up 2 year follow up	Primary outcome/s Child BMIz Secondary outcome/s Child: Dietary intake, physical activity & television viewing and screen time Parent: Perception of own and child’s weight status, dietary intake, physical activity & television viewing and screen time
20. French et al. (2018) USA	To integrate home visiting, community-based parenting classes, primary care provider interactions, and neighborhood connection strategies into a synergistic intervention that targeted low-income, racially, and ethnically diverse parents to prevent obesity among their preschool-aged children	Children aged 2–4 years with a BMI ≥ 50th percentile, from low-income, ethnically diverse families where a parent could speak English or Spanish	Ethnicity: Non-Hispanic white int − 9.1%; con − 16%; non-Hispanic black int − 18.1%; con − 18.6%; Hispanic int − 61.9%; con − 55%; Mixed int − 9.1%; con − 7.8%; Other int − 1.9%; con − 2.6% Gender: Children: (% female): int – 51; con – 51	RCT INT: *n* = 265; CON: *n* = 269; TOTAL: *N* = 534	Children aged 2–4 yr Mean (years) Int – 3.4 (0.7); Con – 3.4 (0.7)	Baseline 1 year follow up 2 year follow up 3 year follow up	Primary outcome/s Child height/weight BMIz Secondary outcome/s Child dietary intake, physical activity
21. Haines et al. (2016) USA	To assess the extent to which an obesity prevention intervention that embeds obesity related messages within a parenting program, compared with controls who received weekly mailings, resulted in a smaller increase in children’s BMI and improvements in weight-related behaviors	Parents and children aged 2–5 years	Ethnicity: Black African American int − 25.2%; con − 19.3%; Hispanic int − 60%; con − 58.1%; white/Other int − 14.9%; con − 22.2% Gender: Parents: (% female): int – 93; con – 93; Children: (% female): int – 51; con – 48	RCT INT: *n* = 56; CON: *n* = 56; TOTAL: *N* = 112	Children aged 2–5 yrs Mean (yrs) Int – 3.6 (1.0); Con – 3.6 (0.9)	Baseline 9 weeks 9 mths	Primary outcome/s Child height/weight BMIz Secondary outcome/s weight-related behaviors (i.e., sleep, TV, active play, and sugar-sweetened beverage intake) Parental feeding behaviors, general parenting skills & confidence
22. Heerman et al. (2019) USA	To test the extent to which dose delivered during a recently completed behavioral childhood obesity prevention was associated with childhood weight outcomes	Children aged 3–5 years from Spanish speaking families with a BMI ≥ 50th percentile	Ethnicity: Hispanic 100% Gender: Children: (% female): int – 54; con – 53	RCT INT: *n* = 59; CON: *n* = 58; TOTAL: *N* = 117	Children aged 3–5 yrs Mean (yrs) Int – 4.3 (0.8); Con – 4.2 (0.8)	Baseline 1 year 2 years 3 years	Primary outcome/s Child BMIz trajectory (12 mths) Secondary outcome/s diet & physical activity, Parent BMI, diet practices, physical activity, self-efficacy for child health behaviors & parenting practices
23. Hughes et al. (2020) USA	To assess the short-term effects of an obesity prevention program promoting eating self-regulation and healthy food preferences in low-income Hispanic children	Parents and children from low-income Hispanic families	Ethnicity: Hispanic 100% Children: (% female) Urban city children (55.4), agricultural Community children (44.8)	Cluster RCT INT: *n* = 136; CON: *n* = 119; TOTAL: *N* = 255	Children aged 3–5 yr Mean (mths) Urban city (53.5 mths); Agricultural community (57.0 mths).	Baseline 1–3 weeks 6 mths 1 year	Primary outcome/s Parents feeding practices, styles & knowledge; Child eating self-regulation, willingness to try new foods & food preferences; Child/Parent BMIz/BMI
24. Esquivel et al. (2016) USA	To measure the effect of a Head Start (HS) policy intervention for childhood obesity prevention	Children aged 2–5 years from low-income families	Ethnicity: Asian 9%; Mixed 62%; Native Hawaiian Americans 23%; white 6% Gender: Children: (% female): 46	Cluster RCT INT: 11 HS classrooms in one cluster area (*n* = 173); CON: 12 HS classrooms in one cluster area (*n* = 176); TOTAL: *N* = 349	Children aged 2–5 years 3 years-old (44%); 4 years-old (47%)	Baseline 1 year	Primary outcome/s Child BMIz, dietary intake (plate waste on lunch tray) & physical activity
25. Natale et al. (2014; 2017, 2021, 2022) USA	To assess the impact of an early childhood obesity prevention intervention on dietary patterns and body mass index percentile over 2 school years (Phase 1 2011-13; Phase 2 2015-18)	Children aged 2–5 years from low-income families which mirrored the ethnic composition of the diverse local population	Phase 1 Hispanic 58%, non-Hispanic black 29%, non-Hispanic white 3%) Gender: Children: (% female): int – 50; con – 49 Phase 2 Hispanic 64%, non-Hispanic black 25%, non-Hispanic white 5%) Gender: Children: (% female): int − 53; con – 51	Cluster RCT Phase 1 INT: *n* = 754 (12 childcare centres); CON: *n* = 457 (12 childcare centres); TOTAL: *N* = 1211 Phase 2 INT: *n* = 465 (12 childcare centres); CON: *n* = 360 (12 childcare centres); TOTAL: *N* = 825	Children aged 2–5 years Phase 1 Mean (mths) Int − 52.6; Con – 44.4 Phase 2 Mean (mths) Int − 42.5; Con – 44.6	Phase 1 T1: Baseline - beg 2011-12 T2 (end of 2011-12 school year) T3 (beg 2012-13 school year) T4 (end 2012-13 school year) Phase 2 T1: Baseline - beg 2015-16 T2 (end of 2015-16 school year) T3 (beg 2016-17 school year) T4 (end 2016-17 school year)	Primary outcome/s Child nutrition and physical activity
26. Petrova et al. (2009) USA	To evaluate the effectiveness of a breastfeeding promotion program in the Women, Infant and Children Supplemental Nutrition Program Participants	Women registered in the WIC program in a maternal care center within a low-income inner-city population.	Ethnicity: White int − 5.8%; con − 5.8%; black int − 1.9%; con − 9 0.6%; Hispanic int − 90.4%; con − 84.6%; Asian int − 1.9%; con − 0% Gender: Parents: (% female): 100	RCT INT: *n* = 52; CON: *n* = 52; TOTAL: *N* = 104	Pregnant women aged 18 yrs or older Mean (yr) Int – 24.8 (5.6); Con − 25.6 (5.6)	Baseline 7 Days 1 mth 2 mths 3 mths	Primary outcome/s Infant feeding practices Secondary outcome/s Maternal knowledge, attitudes, and beliefs regarding breastfeeding
27. Nix et al. (2021) USA	To test whether a preventive intervention was successful in improving protective factors related to overweight and obesity among families living in poverty	Parents and children aged 1.5-3 years from families with incomes below the federal poverty threshold	Ethnicity: Non-Hispanic white 29%; black 29%; Hispanic/Latinx 23% Gender: Children: (% female): 44	RCT Total sample (*N* = 73)	Children aged 18 to 36 mths Mean (mths) − 30.72	Baseline 11 weeks	Primary outcome/s Child healthy eating habits & self-regulation; Parent responsive feeding practices and sensitive scaffolding
28. Reifsnider et al. (2018) USA	To assess whether a parent education intervention, initiated prenatally and provided in the home, would reduce the incidence of infant overweight at age 12 months	Healthy Latina women in the third trimester of pregnancy, aged 18–40 years, with a pre-pregnancy BMI ≥ 25	Ethnicity: Hispanic 100% Gender: Children: (% female): int – 43; con – 41; Mothers: (% female): 100	RCT INT: *n* = 91; CON: *n* = 83; TOTAL: *N* = 174	Pregnant women aged 18–40 yrs Gestation mean (wks) Int − 39.5 (1.1); Con- 39.2 (1.3)	1 wk postpartum 1 mths postpartum 6 mths postpartum 12 mths postpartum	Primary outcome/s Infant weight-for-length Z-scores, Secondary outcome/s breastfeeding status
29. Rosenstock et al. (2021) USA	To assess the impact of a brief home-visiting approach on sugar-sweetened beverage consumption, responsive parenting, and infant feeding practices, and optimal growth through 12 months postpartum	Native American mothers of an infant aged < 14 weeks	Ethnicity: Native American 100% Gender: Children: (% female):52; Mothers: (% female): 100	RCT INT: *n* = 68; CON: *n* = 66; TOTAL: *N* = 134	Infants aged 14 wks or younger Mean maternal age (yrs) 27.4; infant age (mths) − 2	Baseline 4 mths postpartum 6 mths postpartum 8 mth postpartum 9 mths postpartum 12 mths postpartum	Primary outcome/s Sweet and sugar beverage consumption (SSB), responsive parenting & complementary feeding practices inc. initiation. Secondary outcome/BMI Z scores
30. Slusser et al. (2012) USA	To measure over a one-year period whether a parent training based on social learning theory combined with evidence-based interventions to promote optimal nutrition and physical activity will reduce the upward trend of BMI Z-scores in groups of 2–4 year old Latino children living in low-income households	Children aged 2–4 years from low-income Latino families	Mother Birthplace: Mexico int − 82%; con − 78%; Central America int − 14%; con − 20%; United States int − 3%; con − 2% Gender: Children: (% female): int – 56; con – 57	RCT INT: *n* = 80; CON: *n* = 80; TOTAL: *N* = 160	Children aged 2–4 yrs Mean age not provided	Baseline 4 mths 12 mths	Primary outcome/s Child BMIz
31. Pallante et al. (2019) USA	To pilot a parent focused home-based PA intervention implemented through a Head Start center, and to explore changes in children and parent PA, as well as in parents reported social support, social control and regulatory efficacy	Mothers with a BMI ≥ 25 who had children aged 3–4 years	Ethnicity: Latino 100% Gender: Children: (% female): int – 45; con – 67; Parents: (% female): int – 95; con – 100	RCT INT: *n* = 20; CON: *n* = 18; TOTAL: *N* = 38	Children aged 3–4 yrs 3 yrs Int − 25.0%; Con – 16.7%; 4 yrs Int – 75.0%; Con – 83.3%	Baseline 1 week 3 weeks 7 weeks 10 weeks	Primary outcome/s Child BMIz Secondary outcome/s Child physical coordination, physical activity; Parent physical activity, regulatory efficacy, parental influences, & social support
32. Berry et al. (2011) USA	To assist Spanish speaking women from Mexico to manage their weight, prevent progression to Type 2 Diabetes Mellitus and prevent excessive weight gain in their children	Spanish speaking mothers with a BMI > 25 and a child aged 2–4 years	Ethnicity: Hispanic 100% Gender: Children: (% female): 60; Parents: (% female): 100	RCT Mothers INT: *n* = 28; CON: *n* = 28; TOT: *N* = 56 Infants INT: *n* = 28; CON: *n* = 28; TOT: *N* = 56	Children aged 2–4 yrs Mean (yrs) − 3.1 (1.1)	Baseline 9 mths	Primary outcome/s Child & mother BMIz /BMI Adiposity (waist circumference, skinfold) Mother health promoting behaviours Mother eating & exercise self-efficacy, exercise self-efficacy Fasting glucose, insulin, and lipid levels
33. Alhassan et al. (2007) USA	To assess a randomised controlled pilot study to test the hypothesis that increasing preschool children’s outdoor free play time increases their daily physical activity levels	Children aged 3–5 years with no conditions limiting participation	Ethnicity: Latino 100% Gender: Children: (% female): int – 39; con – 28	RCT INT: *n* = 18; CON: *n* = 15; TOTAL: *N* = 33	Children aged 3–5 yrs Mean (yrs) Int − 3.8; Con − 3.5	Baseline Weekly for 3 mths	Primary outcome/s Child physical activity
34. Cepni et al. (2021) USA	To evaluate the feasibility and effects of an intervention playgroup on toddler diet and activity behaviors	Parents with a child aged 12–36 months old	Ethnicity: Hispanic/Latino int − 41.7%; con − 34.6; African American int − 33.3%; con − 11.5; other int − 30%; con − 30.8 Gender: Children: (% female): int – 42; con – 42; Parents: (% female): int – 79; con – 89	RCT INT: *n* = 24; CON: *n* = 26; TOTAL: *N* = 50	Children aged 12–36 mths Mean (mths) Int – 22.3 (6.8); Con – 22.9 (6.5)	Baseline 12 weeks	Primary outcome/s Child dietary intake, BMIz, physical activity, sleep & screen time Parental feeding practices & home/sleep environment.
35. Harvey-Berino & Rourke (2003) Canada	To determine whether participation in an obesity prevention and parenting support intervention reduces obesity prevalence in high-risk Native American children when compared to a parenting-support only intervention	Mothers with a BMI > 25 and with children aged 9 months-3 years that could walk	Ethnicity: Native American 100% Gender: Children: (% female): 56; Parents: (% female): 100	RCT INT: *n* = 20; CON: *n* = 20; TOTAL: *N* = 40	Children aged 9–36 mths Mean (mths) − 21	Baseline 16 weeks	Primary outcome/s Child weight-for-height z and weight-for height percentile Secondary outcome/s Child dietary intake, physical activity, parenting feeding style, maternal self-efficacy & intention to change diet and exercise behaviours
36. Anderson et al. (2005) USA	To assess the efficacy of peer counseling to promote exclusive breastfeeding among low-income inner-city women	Expectant mothers < 32 weeks’ gestation with no medical conditions, who were considering breastfeeding	Ethnicity: Hispanic int − 81.0%; con − 63.9%; black int − 14.3%; con − 2.8%; Caucasian int − 1.6%; con − 12.5%; other int − 3.1%; con − 2.8% Gender: Parents: (% female): 100	RCT INT: *n* = 63; CON: *n* = 72; TOTAL: *N* = 135	Pregnant women aged 18 ≥ yrs < 20 yrs Int – 9.5%; Con – 16.7%; 20–30 yrs Int − 68.3%; Con – 66.7%; ≥30 yrs Int – 22.2%; Con – 16.7%	Baseline 1 mth postpartum 2 mths postpartum 3 mths postpartum	Primary outcome/s Infant feeding practices (exclusive breastfeeding status)
37. Gago et al. 2023 USA	To evaluate whether children in the intervention vs. control experienced greater improvements in BMI z-score and weight-related behaviors.	Children and parents who are enrolled in a Head Start centre for the full school year	Ethnicity: Non-Hispanic Asian int − 13.9%; con − 7.2%; black African American int − 20.3%; con − 41.2%; Hispanic/Latino int − 49.3%; con − 37%; non-Hispanic white int − 12.2%; con − 6.2%; other int – 4.3%; con – 8.4% Gender: Children: (% female): int – 51; con – 50	Cluster RCT 16 Head Start centres INT: *n* = 1631; CON: *n* = 3368; TOTAL: *N* = 4999	Children aged 33 months to 5 yrs old. 2 yrs Int – 11.4%; Con – 11.0%; 3 years Int − 53.5%; Con – 53.9%; 4 years Int – 35.1%; Con – 35.1%	Baseline Fall (Sept-Dec) Spring term (Feb-June) during years 2016–2018	Primary outcome/s Child BMIz, modified BMIz Secondary outcome/s Child dietary intake, physical activity, screen time, sleep duration; weight related parenting practices, parental empowerment
38. Gans et al. 2022 USA	To evaluate the efficacy of the nutrition and physical activity intervention among family childcare providers who receive the Healthy Start intervention with demographically matched control	Family childcare providers who care for 2–5-year-old children.	Ethnicity: Hispanic int − 65%; con − 69.5%; non-Hispanic int − 35%; con − 30.5% Gender: Providers: (% female): int – 53; con – 50	Cluster RCT INT: *n* = 60; CON: *n* = 59; TOTAL: *N* = 119	Children aged 2–5-yrs Mean (mths) – Int – 41.8 (11.8); Con – 41.6 (12.0)	Baseline mth 8	Primary outcome Child dietary quality, physical activity, and sedentary behaviours Secondary outcomes Food, physical activity and screen-time environments and food and activity-related practices of day care setting

**Table 3:** Intervention characteristics of included studies

Author, Year	Intervention	Control	Setting of intervention	Who delivered intervention	Intervention duration (months)	Dose, Intensity and Fidelity (Planned/Actual)	Cultural Adaptation Rank
Toussaint et al. 2021 [95] A Healthy Start programme (AHS) & PLAYgrounds for TODdlers programme (PLAYTOD)	There were 2 intervention programmes: Healthy Start programme (AHS) Early Childhood Education and Care (ECEC) teachers were invited to 3 meetings from a developed AHS curriculum module to improve knowledge and practices on how to be a role model and create a healthy, active, and safe environment for children. PLAYgrounds for TODdlers programme (PLAYTOD) ECEC teachers were invited to 2 x training sessions (1) theory, the importance of physical activity and skill building and (2) coaching on the job session.	Treatment As Usual	Pre-schools	ECEC teachers completed a ‘train the trainer’ course to become coaches to train colleague teachers. Certified PLAYTOD trainers	9 months	AHS 3 × 2-hour face-to-face meetings were delivered to teachers PLAY-TOD Teachers attended 2 × 2-hour face-to-face training sessions were delivered to teachers (two weeks apart) Actual dose and fidelity were not reported.	Minimal
Messito et al. 2020 [48, 72], Gross et al. 2016 [47] The StEP Programme	Family centred, strengths-based program to promote healthy behaviours. The main components included: (1) prenatal nutrition counselling (2) postpartum lactation support (3) nutrition and parenting support groups coordinated with pediatric visits.	Treatment As Usual	Pediatric Hospital	Bilingual (English/ Spanish) registered dietitians & certified lactation counsellors	36 months	15x sessions were delivered to parents inc. 2 individual (third trimester; postpartum) and 13 group sessions at 1, 2, 4, 6, 9, 12, 15, 18, 21, 24, 27, 30, and 33 months. Fidelity was assessed for 74% of sessions and found that 97% provided all curriculum components. Mean number of sessions attended was 7.75 (4.5) (of possible 15). Dose-dependent effects were found.	Comprehensive
Natale et al. 2014 [53] HI-HO Programme	Family based education programme focused on increased physical activity and fresh produce intake, decreased intake of simple carbohydrate snacks, and decreased screen time. *Teacher Component*: deliver curriculum based on modified version of Hip-Hop to Health + weekly technical assistance with HiHO specialist to support implementation. *Parent Component*. A monthly educational dinner in which nutrition, perceptions on weight, physical activity and screen time were discussed (tailored and delivered by dietician), monthly newsletters, and at-home activities. *Centre-Based Modifications Component*: Policies to increase physical activity (1 h > per day & decrease TV viewing < 60 min 2 x pw) & improve healthy eating (Nutritionist modified menus, drink policy advocating water & 1% milk + healthy snack policy)	Attention control. Received similar levels of training & assistance with a safety education curriculum.	Childcare setting	Teacher component: Teachers with Hi-Ho advisor support Parent component: Registered dieticians with same cultural background Centre based: The setting/staff with HiHO advisor and nutritionist list support	6 months	Teacher component: 2 x training sessions Parent component: Monthly x 6 educational dinners and at home activities Actual fidelity not reported. Dose-dependent effects were found.	Comprehensive
Vaughn et al. 2021 [73] Healthy Me, Healthy We (HMHW)	Social marketing campaign delivered across a school year. 4 × 6-week units which included: (1) Play More, Get Thirsty (2) Together is Better (3) Let’s Move and Find our Snacking Groove (4) Try a New Thing in Spring Each unit promoted healthy eating & physical activity goals through classroom and home which were concluded with a celebration event ‘Healthy We’ family guide magazine provided to parents to introduce unit goals, outline benefits of adopting healthier behaviors, and provide suggestions about what parents can do to establish healthy habits and fulfill their Fit Family Promise.	Wait list control	Childcare Centres/ Home	Childcare centres, providers & parents Childcare directors/ teachers received 2 x training sessions & several visits by the HMHW interventionist. All participated in the 2 prescribed trainings received the 3 planned assistance visits and were provided with all campaign materials.	8 months .	Launch & celebration event 4 × 6-week units Newsletters Poster, activity cue cards (to inform 2 x classroom activities pw), and ‘Our Turn Cards’ (sent home to parents after an activity to prompt corresponding at-home activity). Parents received family guide at beginning of each unit) and an Activity Tracker to track completion of at-home activities. Less than half of parents received the Family Guides (66% for unit 1, 48% for unit 2, 45% for unit 3, 50% for unit 4). 55% parents did not receive Our Turn Cards, with 9% reported receiving < 4.	Minimal
Tomayko et al. 2016 [74] Healthy Children, Strong Families (HCSF)	Obesity prevention toolkit which consisted of 12 x culturally appropriate lesson plans, activities and resources delivered in the participant’s home. Each lesson addressed one of four target areas: (i) eat more fruits & vegetables; (ii) consume less soda & added sugar; (iii) become more active; and (iv) watch less TV.	Received the same HCSF toolkit via mail with activities self-guided.	Home based	Trained home mentors who are tribal members and/or individuals with long-standing employment within community.	24 months	12 × (60 min) monthly family home visits informed by an intervention toolkit Families received mthly newsletters in Year 2 alongside mthly group meetings to support behaviour changes developed during Year 1. The non-mentored families received the toolkit via email and the newsletters in Year 2. Actual dose and fidelity not reported.	Moderate
Wasser et al. 2020 [75] Mothers & Others	Obesity Prevention Group (OPG) Received home visits, information toolkit, and four newsletters designed to provide anticipatory guidance (AG) and support for enactment of six targeted infant feeding and care behaviors: breastfeeding; adoption of a responsive feeding style; use of non-food soothing techniques for infant crying; appropriate timing and quality of CF; minimization of TV/media and, promotion of age-appropriate infant sleep.	Injury Prevention Group (IPG) Attention control. Received same number of home visits, a toolkit, and newsletters although based on an injury prevention programme published in AAP Bright Futures.	Home based	Peer Educators (PE) Women with a MS/MPH degree or BS/BA degree in a health-related field + 2 + yrs experience providing counseling. Breastfed own children & received over 100 h of training.	15 months	8 x home visits 6 x home visits were delivered by a PE at 30- and 34-weeks’ gestation and 3-, 6-, 9-, and 12-months postpartum. Intervention families could also receive up to two additional home visits by an International Board-Certified Lactation Consultant (LC) after hospital discharge. Dose-dependent effects were found.	Minimal
Annesi et al. 2013 [69–71] Start for Life	30 min of daily structured physical activity which incorporated gross motor skills and behavioural skills training. Behavioural skills included long- and short-term goal setting, self-monitoring of incremental progress and acknowledgement of physical achievements via logs and certificates. Self-regulation via (i) specific performance feedback, (ii) setting graded tasks, (iii) providing specific encouragement and (iv) providing information about the behaviour-health link. Brief stress reduction techniques were also incorporated.	Usual Care	Pre-schools	Pre-school teachers received general guidelines of age-appropriate physical activities for structured and unstructured physical activities & also received additional 4-h training of cognitive-behavioural methods	8 weeks	30-minute sessions delivered to children daily during school Actual dose and fidelity non provided.	Minimal
Barkin et al. 2012 [76] Salud Con La Familia (Health with the Family) program.	Weekly 90-minute skills-building sessions for parents and preschool-aged children designed to improve nutritional family habits, increase weekly physical activity, and decrease media use (sedentary activity).	A brief school readiness program was conducted as an alternative to the active intervention.	Community based (Recreation centre)	Trained bilingual facilitator	3 months	Parents and children in the intervention group were provided a wkly 90-min skill building sessions across 3 mths (*n* = 12). Parents and children in control group met 3 times (60 min) over 12-week study period. Actual dose not provided. Observations for 3 sessions for each condition revealed 100% of intended messages were fully discussed & all planned activities occurred. Intervention content was never delivered in control sessions or vice versa.	Comprehensive
Barkin et al. 2018 [49] Heerman et al. 2020 [50] Growing Right Onto Wellness (GROW)	The intervention was split into phases: (1) 12-week intensive phase with weekly skills-building sessions; (2) a 9-month maintenance phase with monthly coaching telephone calls; and (3) a 24-month sustainability phase providing frequent cues to action for healthy family behaviors. Intervention content included skills building for parents and children regarding nutritional choices, physical activity habits, use of family & built environment, engaged parenting, healthy sleep, & reduced media time. Each week (intensive phase) or month (maintenance phase), participants created a self-defined goal about family health behaviours targeted in the intervention (diet, physical activity, sleep, media use, engaged parenting).	GROW Smarter Attention control. Received school-readiness program developed and delivered by the local library	Community based (community centre)	Trained facilitators	36 months	Intervention (1) 12 wks weekly 90 min skills building sessions (via person/telephone) delivered to families (2) 9 months – mthly coaching telephone calls 24 months – receive frequent contacts (mail/calls/text) Additional coaching call if child weight increased or if they remained obese at data collection time point. The mean dose received among intervention participants was 92% for the intensive phase, 87% for the maintenance phase, and 85% for the sustainability phase. The control group received 6 × 30-minute group-based activities. Fidelity of curriculum was 99% across the 36 months.	Minimal
Beck et al. 2017 [77]	A healthy beverage intervention where participants received recommendations, knowledge which was tested and completed a role play exercise alongside goal setting.	Attention control. Interactive reading intervention.	Clinic (outpatient pediatric)	Intervention facilitators	2 weeks	Parents attended a 20 min educational intervention delivered by a trained facilitator. Actual dose/fidelity not reported.	Moderate
Black et al. 2021 [78] ToT-TOPS & Mom-TOPS	2 x intervention programmes ToT-TOPS Responsive parenting & maternal lifestyle emphasised parents’ role in recognising/responding to signals, behaviour management, soothing without relying on food promoting toddler autonomy & providing opportunities for healthy meals/ physical activity. Mom-TOPS Maternal lifestyle intervention focused on maternal diet and physical activity with no mention of responding to toddler diet, physical activity /behaviour.	Safe-TOPS Attention control intervention that focuses on home safety	Women, Infants, and Children (WIC) clinic setting	Health educators with Masters-level education. Supervision by a psychologist, Attended a 6 wk-session training programme based on manualised intervention and motivational interviewing.	4 months	8 x sessions held bi-weekly over four months. 4 x group sessions three individual telephone coaching sessions Final group session > 90% of the planned topics for each session were delivered across both groups Attendance across intervention sessions did not differ between groups (56–60%). 71% attended at least one session.	Minimal
Bürgi et al. 2012 [66] Ballabeina	Intervention was based on lifestyle behaviours: physical activity, nutrition, media use and sleep. The study was designed at individual and environmental levels, focused on promoting changes in education, attitudes, and behaviour, and providing social support.	Treatment as usual including the regular school curriculum that includes 1 × 45 min PA lesson pw	Pre-Schools	Teachers Attended 2 × 3 h workshops Supported by visits of health promoters & hands-on training	Full school year	Physical activity program consisted of 4 × 45 min sessions pw. Health promoters delivered one lesson pw reduced to 2x mthly after 4 months. All other lessons were delivered by the regular preschool teacher. 22 lessons on healthy nutrition, media use, and sleep. Parents participated in 3 x interactive evenings about promotion of PA, healthy food, limitation of TV use and the importance of sufficient sleep. Actual dose and fidelity not reported	Minimal
Davis et al. 2016 [51] Cruz et al. 2016 [52] CHILE	The intervention had six components: (1) a nutrition & physical activity curriculum with repeated opportunities to taste a new fruit/vegetable and to add 30 min of physical activity to daily class activities (2) quarterly professional development training for teachers and catering staff. Training sessions were given prior to each of 8 nutrition modules, over a 2-year period. (3) a component focused on integrating policy and behaviour change in food purchasing, preparation, and serving by service staff (4) a family component consisting of take-home materials (newsletters/ activity cards) nutrition and physical activity and family events reinforcing these messages twice during the school year (5) a local grocery store component with the goal of increasing availability and visibility of healthier food options and providing recipes and nutrition-related information to families while shopping (6) local healthcare providers to emphasise healthy eating and physical activity during routine patient visits and invited health professionals to attend CHILE family events to show support for the intervention.	Treatment as usual	Head Start Pre-school setting	Healthcare providers	24 months	60 min unstructured play 30 min structured play daily. Quarterly sessions for staff (*n* = 8) Family events were stand-alone events (*n* = 24) in first year and 16 in the second year. Of the 2688 potential nutrition lessons (8 lessons × 8 modules × 42 classrooms across 8 HS centres), 1935 (72%) were reported completed. Completion rates increased steadily in the first year of the intervention, with 63% of lessons completed in Year 1, and 81% of lessons completed in Year 2. All professional development sessions planned were held and lasted 2–3 h	Comprehensive
Fernandez-Jimenez et al. 2019 [79] FAMILIA	Multicomponent intervention (promoting healthy diet, increasing physical activity, understanding human body, and managing emotions) which included curricular units delivered by the preschool teacher. Strategies were also used to develop child health behaviours with parents which included invitations to educational meetings alongside a minimum of 11 family health activities which were provided by the teacher.	Waitlist control	Pre-schools	Pre-school teacher	4 months	Intervention included a minimum of 37 h for children & 12 h for parents/caregivers) with a total of 50 h of educational activities. 32% of children (*n* = 96) received > 75% of the educational modules (high-adherence group) while 39% (*n* = 120) received between 50% and 75% of the modules (intermediate-adherence) and 29% (*n* = 88) received < 50% of modules (low-adherence). Dose effects were reported.	Moderate
Fiks et al. 2017 [80] Grow2Gether	Intervention involved online group activities which begun 2 months prenatally to facilitate mothers’ bonding before delivery, until infant was aged 9 months. Included 2 x in person group meetings (1) prenatal for introductions and setting group ground rules (2) at infant age 4 mths 4 x peer groups were formed (9–13 women) on infant due date. Curriculum delivered focused on infant feeding practices, sleep, positive parenting, activity, parenting expectations, infant cues and calming), and maternal well-being. A Facebook group was structured around a video-based curriculum and encouraged participant interaction. Videos featured mothers and infants (many from the same community as the participants) demonstrating behaviours and discussing topics related to healthy infant growth. Information presented in videos was also provided to the group.	Treatment as usual	Hospital clinics and online	Psychologist specialising in obesity treatment.	11 months	2 x in person mother group meetings Mothers provided wkly videos from start until infant age 6 mths, then bi-weekly until 9 months. All intervention participants successfully joined & posted in assigned FB group. Group members posted a mean 30 x per group per week, which is more than twice the rate of posting that was defined as ‘‘active engagement’’. Participants were most active in the groups around the perinatal period. Prenatal curriculum 1953 posts across the 4 groups); 0 to 3 mths (1802); 3 to 6mths (1074); 6 to 9 mths, when curriculum was posted less (533)	Moderate
Fisher et al. 2019 [81] Food, Fun, and Families (FFF)	Weekly sessions that focus on (1) connecting with children and building lasting family bonds at mealtimes; (2) preventing hyperactivity and tooth decay; (3) using feeding to teach children life lessons about limit setting and structure; and (4) being responsive to children during mealtimes. Sessions began with group discussion of progress from previous weeks’ goals and collective problem-solving (SHARE; 25 min), followed by presentation of new content and interactive demonstrations (GROW; 25 min), and a discussion of the next week’s goals (GO; 5 min).	Delayed treatment control	Women, Infants, and Children (WIC) clinic setting	2 x graduate-level interventionists who received training from clinical psychologists with expertise in behavioral interventions	12 weeks	12 x weekly 60 min face to face sessions Mean attendance was 6.4 (SD = 4.2) out of 12 sessions, with 18 (30.5%) mothers attending fewer than 25% of sessions, 4 (6.8%) attending 25–49% sessions, 14 (23.7%) attending 50–74% and 23 (40%) attending 75% or more of the sessions during the 12week intervention	Minimal
Fitzgibbon et al. 2002; Stolley et al. 2003; Fitzgibbon et al. 2005; [57–59] Hip-Hop to Health Jr Phase I	Weight Control Intervention (WCI) A curriculum was delivered with a different topic introduced each week. Thrice weekly lesson plans were used to structure the sessions: (1) 20 min lesson which introduced healthy eating /exercise concept with an activity. Incorporated puppets which led the children through various activities and adventures. (2) 20 min of teacher led physical activity which including 5-minute warm-up, 10 min of aerobic activity, and a 5-minute cooldown. Parents received weekly newsletters which reflected the curriculum which also had sections on healthy eating and exercise with a homework assignment.	General Health Intervention (GHI) Attention control. Received curriculum related to general health concepts (not diet/physical activity). Received wkly newsletters that mirrored the GHI.	Head Start preschools	Trained early childhood educators.	14 weeks	Intervention 14-week (40 min, 3xwkly) + weekly newsletters Teachers reported completing a mean of 26.6 (SD = 1.9) from a total of 28 nutrition lessons and 27.1 (SD = 1.7) from a total of 28 exercise lessons. Parents completed a mean of 5.0 (SD = 4.5) of 13 homework assignments. Control 14-week (20 min, 1 x weekly) + weekly newsletters	Comprehensive
Fitzgibbon et al. 2011 [61] Kong et al. 2016 [62] Hip-Hop to Health Jr Phase II	Teacher Delivered Weight Control Intervention (TD-WCI) The curriculum was similar to that reported in Phase I except the curriculum was delivered across two weekly sessions with the option of including a third session if they chose. Each session in line with Phase I included a 20-min lesson related to healthy eating and exercise, as well as a 20-min physical activity component. Sessions incorporated a CD for teachers to play for their students which included songs and raps with two fully scripted exercise routines. Parents received a weekly newsletter that paralleled the children’s curriculum in content and included homework. Parents received a financial incentive for each homework assignment completed/returned.	Teacher delivered general health intervention control group (TD-GHI) Attention control. Received curriculum related to general health concepts (not diet/physical activity). Parents received a weekly newsletter that mirrored the weekly theme of the school-based curriculum but were not asked to complete homework assignments.	Head Start preschools	Pre-school teachers Teachers received an initial 3-hour training session. Intervention teachers received three in-school training sessions delivered by intervention coordinator with one in school session for the control teachers. Intervention coordinator met weekly with TD-WCI teacher and monthly with TD-GHI teacher over the course of the 14 weeks.	14 weeks	14-week (40 min, 2xwkly) + weekly newsletters Teachers reported completing or partially completing a mean of 26.6 (SD = 1.9) of the 28 nutrition lessons and 27.1 (1.7) of the 28 exercise sessions. Seven of the 18 teachers (39%) taught all of the nutrition lessons, and 10 (56%) of the teachers completed all of the exercise sessions. The Hip-Hop to Health CD was used in almost all (94%) of the exercise sessions. Twenty (7%) of the parents completed all 13 assignments. One hundred fifteen parents (37%) completed more than half of the homework assignments, and 238 (78%) completed at least one assignment.	Moderate
Fitzgibbon et al., 2013 [57, 63] Family-Based Hip-Hop to Health Jr Phase III	Family-Based intervention (FBI) Child Curriculum The curriculum was similar to that reported in Phase I with the inclusion of a Spanish language CD to supplement the curriculum. Parent curriculum Encouraged to attend six weekly 90-min classes that included 60 min of interactive instruction on healthful eating and family exercise plus 30 min of moderate physical activity (e.g., salsa aerobics, walking group). Weekly newsletters containing culturally adapted information that paralleled the school-based component.	General Health Intervention (GHI) Children Attention control. Received curriculum related to general health concepts (not diet/physical activity). Parents Weekly newsletters that paralleled the GHI curriculum.	Early child education setting	Bilingual/bicultural educators	14 weeks	A total of 23 out of 61 (38%) intervention group parents who completed a baseline attended at least one of the six classes offered.	Comprehensive
French et al. 2018 [65] Now Everybody Together for Amazing and Healthful Kids (NET-Works)	Three components: (1) home visiting, (2) community-based parenting classes (3) telephone check-in calls. Referrals to community resources for healthy foods and physical activity opportunities were embedded in the home visiting and parenting class components.	Providers discussed child BMI with parent at the annual well-child visit, by using a study-provided pamphlet with the child’s BMI percentile and messages about healthful eating/PA for the child. Parents also received quarterly postcards that focused on child development and school readiness.	Home and Community based	Trained professionals with a minimum of a bachelor’s degree + several yrs experience working with families	36 months	Home visits were approx. 1 h in duration across monthly intervals with telephone check-in calls between home visits. Parenting classes were held weekly for 12 weeks in the communities where the families resided. Planned intervention dose was the same across all 3 intervention years. Families received an average of 35.4 contacts over 3 years (15.5, 10.9, and 8.6, in years 1, 2, and 3, respectively). Families received an average of 18.3 home visits (of 36; 50% of intended dose), 9.3 parenting classes (25% of the intended dose), and 7.4 check-in calls (58% of intended dose).	Minimal
Haines et al. 2016 [82] Parents and Tots Together	Family-based obesity prevention intervention Group based sessions (9–13 participants). Each session focused on a weight-related behavior addressed in the parent program and included: (a) reading a related book, (b) an activity such as yoga, music/dance, and (c) preparing a healthful snack. Children were given items, such as balls or water bottles.	Families were mailed publicly available educational materials on promoting healthful behaviors among preschoolers each week for 9 weeks.	Community based (community health centre)	Facilitators (*n* = 3) received 8 h of training on the curriculum and group facilitation process	9 weeks	9 weekly 2-hour group sessions 52% attended 6 or more of the 9 sessions, 11% attended 3 to 5 sessions, and 37% attended 2 or fewer of the sessions	Minimal
Heerman et al. 2019 [64] The COACH Trial	The intervention consisted of two phases: (1) intensive in-person phase (2) maintenance phone-call phase. Phase 1: consisted of weekly group sessions for 15 weeks which were based on a curriculum to build parent and child agency for health behaviors through a personalised approach. Children participated in a concurrent session designed to mirror the adult intervention. These sessions included: (1) an interactive didactic component, (2) a hands-on connected activity, (3) stretching and physical activity, and (4) a review. Children joined the parent session for shared interaction around material learned in the session and a shared meal. As a part of each session, the parents received written materials to reinforce key messages.	Attention control. Received 2xmthly school readiness curriculum for 3 months.	Community based (local Park and recreation community center)	Natively bilingual health Coach (unsure of training)	6 months	Intervention 15 wkly group sessions (90 min) followed by 3 mths of twice-mthly health coaching calls which lasted 20–30 min. Control Sessions delivered twice a month for 3 months. Mean number of intensive phase sessions completed was 11.0 of 15 (SD = 5.5), the mean *n* of intensive phase in-person sessions was 6.5 (SD = 4.9), & mean *n* of maintenance phase phone sessions was 3.6 of 6) (SD = 2.6).	Comprehensive
Hughes et al. 2020 [83] Strategies for Effective Eating Development (SEED)	A 7-week program curriculum delivered during weekly parent and child sessions (held separately but simultaneously) and a family session (parent and child together). The program included parental strategies to promote appropriate child portion sizes, structure, and routines in the family environment, and dealing with outside influences on child eating.	Treatment as usual	Childcare educational setting (Head Start)	Facilitators matched the ethnicity of the participants	7 weeks	7-week with 1 lesson/wk (a total of 7 lessons) The mean number of lessons attended by mothers in the prevention group was 5.17 out of 7 (SD = 2.11): 35% of mothers attended all 7 lessons, 24% attended 6, 14% attended 5, 7% attended 4, and around 5% mothers attended 3<.	Comprehensive
Esquivel et al. 2016 [68] Healthy Living Programme (HLP)	The implementation of a wellness policy for childhood obesity prevention. The intervention focused on healthy eating practices, resources for the classroom for classroom nutrition and physical activity. The intervention included: (1) Monthly employee wellness activities for teaching staff (2) resources for classroom nutrition and PA activities – monthly lessons (3) monthly family newsletter	Treatment as usual; delayed intervention	Childcare educational setting (Head Start)	N/A	7 months	Monthly employee activities Monthly family newsletter Actual dose and fidelity not reported.	Moderate
Natale et al. 2021 [55]; 2022 [56]; 2017 [54] Healthy Caregivers-Healthy Children	This was a multi component environment policy intervention which included the three following components. (1) teachers/parents (role modelling)/children curriculum which were related to core less plans. (2) snack, beverage, physical activity, and screen time policies. (3) menu modifications.	Received injury prevention curriculum which provided parents and teachers with safety information.	Childcare educational setting	Phase 1: Research team Phase 2: a train-the-trainer approach was used.	24 months	Both arms were followed and/or received treatment for two school years (approximately 9 months each), The parent/teacher curriculums consisted of 6 mthly workshops. Actual dose and fidelity not reported.	Comprehensive
Petrova et al. 2009 [67]	All women received in addition to routine ‘usual’ care breastfeeding education during remainder of pregnancy and postdelivery support. Prenatal 2x face to face individual educational sessions at 2–4-week intervals which corresponded with their clinic visit. Any missed visit would carry over to their next clinic visit. During each visit women would meet the lactation consultant who were educated on benefits of breastfeeding, encourage exclusive breastfeeding and delay introduction of formula. Translated educational material was also provided to women. Postpartum After hospital discharge - End of first/second week/alongside month one and month 2. Lactation consultant provided breastfeeding support/education via phone or at hospital. Sessions centred on answering any questions, demonstrate techniques to resolve any problems and promote maintenance of breastfeeding. Home visits provided where there were issues related to pain/discomfort when breastfeeding.	Usual practice Routine breastfeeding support and education during pregnancy and postpartum. A lactation consultant would support any woman who has any breastfeeding problems during hospital stay. Variability of routine breastfeeding education was similar in intervention and control groups.	Hospital	Hospital lactation consultant	4 months	Prenatal 2 x face to face-to-face individual educational sessions (15–20 min) at 2–4 week Postnatal 3 × 15-minute consultation (week 1-Month 2) Actual dose and fidelity not reported	Minimal
Nix et al. 2021 [96] Recipe 4 Success	The intervention consisted of 10 consecutive weekly lessons, as part of the families’ regular Early Head Start home visits. Each ‘Recipe 4 Success’ lesson included some didactic information with most of each lesson focused on active coaching of carefully structured and sequenced food preparation activities involving 3 to 6 ingredients provided.	Usual practice	Home based	Early Head Start home visitors. Home visitors completed one day of training and received a detailed manual. Facilitators were encouraged to engage with a supervisor/ investigators for regularly either via call/e-mail.	10 weeks	10 consecutive weekly lessons Each lesson was 45 min of total 90-min home visits. Intervention participants received different content for the intervention period but did not receive extra or longer home visits. Actual dose/fidelity not reported.	Minimal
Reifsnider et al. 2018 [84]	Promotoras delivered prenatal and postpartum home visits once before delivery and at ages one and two weeks, and two, four, six, nine, and 12 months. During visits, the promotoras discussed infants’ growth, health, development, sleep and play/exercise activities. Promotoras counseled on feeding at each visit regardless of the infant’s growth. Mothers were encouraged to breastfeed and/or to avoid giving excess formula or adding sugar or solids to the bottle. A lactation consultant visited mothers who reported problems with breastfeeding. At each visit mothers in the intervention group received a small gift such as a children’s book.	Treatment as Usual	Home based	Bilingual promotoras (Spanish/ English) women experienced in providing health education to the local population. Received 320 h (40 days) of training based on the American Academy of Paediatrics, Bright Futures.	12 months	Home visits (*n* = 8) Actual dose/fidelity not reported.	Moderate
Rosenstock et al. 2021 [85] Family Spirit Nurture (FSN)	A brief home-visiting approach using was used based on the Family Spirit Nurture (FSN) curriculum. Participants were provided 6 lessons delivered 3 to 6 months post-partum by which covered optimal infant feeding practices, responsive feeding, avoiding sugar and sweetened beverages, optimal complementary feeding practices, and whole family healthy eating practice. Each 45-minute lesson included a warm-up, lesson content and activities, and question and answer period, referrals as needed, and summary handouts.	The control group received 3 educational lessons, at 3-, 4-, and 5-mths post-partum on injury prevention. Control lessons used the same format as intervention-based lessons.	Home based or other private locations.	Navajo paraprofessionals	3 months	Intervention 6 lessons (45 min) Control 3 lessons (45 min) Actual dose/fidelity not reported.	Moderate
Slusser et al. 2012 [86]	Participants were provided with nine module sessions of parent training which were designed to incorporate healthy nutrition and physical activity messages within existing field-tested parent training modules. The content for the healthy nutrition and physical activity messages was based on the “bright Futures in Practice: Physical Activity” and “bright Futures in Practice: Nutrition” health supervision tools and the “traffic Light” diet, the AAP expert work group’s recommendations, and the philosophy of internal regulation related to eating.	A wait list control group were not administered the parent training modules during the study period but were instead offered the classes after the one year follow up. The control group continued to receive treatment as usual and received a standard nutritional informational.	Family health clinics; community settings; childcare education settings	All classes were facilitated by one bilingual social worker who was trained to follow the outline and to maintain consistency between cohorts.		There were 9 × 90-minute sessions. Actual dose/fidelity not reported.	Comprehensive
Pallante et al. 2019 [87]	The Active Playtime intervention consisted of three aspects: (1) parent training to increase knowledge to provide PA for their child and parent skills (self-regulatory and leading PA); (2) provision of play and sports equipment in conjunction with an activity sheet containing examples of games and activities appropriate for children ages 3–5 years; and (3) facilitation of parent-child playdates.	The control group did not receive the training, activity sheets, or playdates. Only used play equipment bi-wkly	Childcare educational setting (Head Start)	Research team (not the same language as some participants) – training not provided	7 weeks	One training session (week one) and two parent – child play dates (week three and week seven). Sessions were 1–2 h long. Actual dose/fidelity not reported.	Minimal
Berry et al. 2011 [88]	The intervention group received weekly sessions which incorporated an education training session which were followed by physical activity. (1) Education and coping skills training classes (2) Nutrition and exercise education and coping skills (3) Exercise classes Women received a 45-minute exercise class, which included stretching, walking, and cardio kickboxing. While the mothers were in class, the children received 1 h and 45 min of free play in a playground at the church.	Wait list control group	Community setting (church)	Community health educators and promotoras	12 weeks	12 x weekly 60-minute training classes (education and coping skills training) followed by 45 min physical activity. Actual dose/fidelity not reported.	Comprehensive
Alhassan et al. 2007 [89] RECESS	RECESS During normal school days preschoolers were scheduled for approximately 60 min of total recess time per day (30 min in the morning and 30 min in the afternoon). During this time participants were taken to the playground where they were free to engage in any form of play. On two days the intervention group received a total of 60 min of additional recess time per day, divided into two 30-minute time blocks (one in the morning and one in the afternoon). The children’s normal recess time was also unstructured.	The control group followed their normal preschool schedule and usual classroom activities and recess time (60 min).	Childcare Education setting	n/a	3 months	Children received daily 60 min. of additional recess time per school day (30 min. in the morning and 30 min. in the afternoon) on two days. Actual dose/fidelity not reported.	Minimal
Cepni et al. 2021 [90] FUNPALs	FUNPALs Playgroups involved physical and snack activities, delivery of health information, and positive parenting coaching. Community health workers delivered sessions according to the lesson plans: (1) an opening welcome song that included each child’s name to enhance a sense of belonging, (2) five short moderate-to-vigorous physical activities. (3) in vivo parent coaching breaks (4) healthy snack preparation activity and a yoga activity & good-bye song.	The control group involved group health education for parents only.	Community setting (community health and fitness centre	Trained community health workers	10 weeks	Parents and children attended 10-weekly 90-minute sessions of the FUNPALs Playgroup. Participants attended an average of 5.5/10 sessions (SD 3.2). No participants withdrew from the FUNPALs Playgroup, but 3 participants (12.5%) never attended the playgroup.	Moderate
Harvey-Berino & Rourke, 2003 [91] An obesity prevention plus parenting support (OPPS)	Obesity Plus Parenting Support (OPPS) As similar to the PS (control) condition however the intervention groups lessons focused exclusively on how improved parenting skills could facilitate the development of appropriate eating and exercise behaviours in children.	Parenting support (PS) Program based on a parenting curriculum. Included 11 different lesson topics covered over 16-week period. Peer educators instructed to not discuss eating /exercise behaviour or limit conversations	Home based	Peer educator received intensive 120-hour initial training conducted by the PI and a family therapist/ parenting consultant. Received mthly staff development sessions. During intervention peer educator met with the PI wkly to review cases.	16 weeks	16 weekly lessons at home visits (time of visits not reported). Actual dose/fidelity not reported	Minimal
Anderson et al. 2005 [94] Peer Counseling on Exclusive Breastfeeding	Peer counselling group (PC) Women were offered 3 prenatal home visits, 9 postpartum home visits, and daily in-hospital visits during postpartum hospitalisation, from the assigned peer counsellor. Telephone counselling as required. This was in addition to the routine breastfeeding support and education received by the control group.	Received conventional breastfeeding education prenatally. On delivery women received routine support and assistance if they experienced any issues breastfeeding. Participants were treated similarly to all private paying patients who delivered their babies at the Hospital.	Home and hospital based	Peer counsellors (2 x mothers who breastfed a child > 6 mths & motivated to help other mothers. Received 40-hour breastfeeding counselling training facilitated by an international board-certified lactation consultant. Observed for 2 mths by lactation consultant.	3 months	3 pre-natal home visits 9 post-partum home visits Week 1: 3 visits Week 2: 2 visits Week 3–6 1 visit Daily in-hospital visits during postpartum hospitalisation . Telephone counseling as required. Coverage by the peer counselors ranged from 56 (88.9%) of 63 for the prenatal home visits to 40 (63.5%) of 63 at week 6 post-partum. Approximately 3% of mothers in the CG reported having received breastfeeding counseling from the existing hospital’s peer counseling service. Four mothers in the PC declined to see the study peer counselor.	Moderate
Gago et al. 2023 [92] Communities for Healthy Living (CHL)	Parents Connect for Healthy Living (PConnect) 10-week parent-staff co-led program Topics include health habits, mindfulness, advocacy, communication, and parenting strategies. Media resources; print materials, social media, web-tools. Nutrition support staff training Talking points, revised BMI (Health and Growth) letter updates	Passive enrollment All clusters were allocated to receive the intervention at one of three intervention start times (years 1–3)	Childcare educational setting (Head Start)	Program sessions were co-led by a Head Start parent and staff member. Head Start parent facilitators match cultural context of centre	2 Years	PConnect 2 h x 1 session per week for 10 weeks Wkly activities for parent/child Wkly content posted to a closed parent Facebook group to encourage discussion. Enhanced nutrition support 1 x primer letter & revised Health and Growth Letter per year; Staff training of staff to support interactions. Media resources Monthly brochures, posters & flyers Year 1: 1400 flyers to 542 families; 56 Head Start nutrition staff trained (71–100% of eligible staff) and 36 PConnect sessions were implemented; Year 2: 5000 flyers to 765 families; 132 staff trained (47–100% of eligible staff) and 72 PConnect sessions implemented. Media campaign and enhanced nutrition support were only implemented for six out of planned 10 months with no PConnect in year 3 due to COVID-19. Low dose - insignificant difference in empowerment/ parenting practices; High dose - significant increase in empowerment	Comprehensive
Gans et al. 2022 [93] Healthy Start/Comienzos Sanos	Family Child Care Homes (FCCH) intervention (1) monthly telephone calls from a support coach (peer counsellor) using brief motivational interviewing (2) Educational print (newsletters/reports) and videos tailored to the needs /interests 3) group support meetings (4) active play toys.	Active control Also receives the four components of intervention however FCCPs are assigned a support coach who have been trained in literacy/reading readiness. Group sessions are held separate	Family child Care Homes (FCCH)/ family day care	Community Health Workers trained in using Motivational Interviewing (16 h training)	8 months	In person baseline visits with support coach 1 x monthly telephone calls (7 in Total) 1 x monthly newsletter and video (8 in total) Group meetings every 6 weeks All completed in-home coaching visit; 82% completed 7 x phone calls; over 80% of FCCP (83%) reported reading all 8 tailored newsletters; 58% reported watching all 8 tailored videos with 89% watching at least half of them; FCCP reported using active toys median of 7 x pw, and activity cards a median of 1 x pw; 60% of FCCP watched all six of the toy video segments with 69% watching at least half; 58% of FCCP attended no group meetings, 12% attended at least half group meetings.	Comprehensive

## Results

### Search results and study selection

A total of 69,945 references were identified. After title and abstract screening, 516 studies were retrieved and subject to full-text review, with 34 intervention studies included in the final review. Four further studies were identified following supplementary searching. A total of 38 interventional studies were published across 49 papers (Fig. 1). There were five intervention studies which were published in multiple articles: Starting Early Programme, with outcomes at three months [47], ten months [48], and three years [48] reported separately; Growing Right Onto Wellness trial, reported outcomes [49] and dose [50] separately; Child Health Initiative for Lifelong Eating and Exercise (CHILE) trial which reported weight and dietary intake [51] and physical activity outcomes separately [52], and finally Healthy Caregivers–Healthy Children intervention which was delivered across two phases, phase 1 (2010-12) [53, 54] and phase 2 (2015-17) [55, 56], which are reported separately. The Hip Hop to Health Jr trial [57, 58] was initially developed and delivered over five years across two waves: 1999–2001 [59] and 2001 and 2003 [60] (Phase I). Based on the original curriculum, the intervention was slightly adapted and delivered in 2005–2009 [61, 62] (Phase II) alongside a family-based Hip-Hop to Health Jr trial [63] (Phase III). Given the subtle changes to the intervention content and cultural adaption, we report the intervention phases separately.

### Study quality

Twenty studies (53%) were appraised as high quality, satisfying 80% or more of the appraisal criteria; five of which met al.l criteria; these included Healthy Caregivers-Healthy Children [54, 56], The COACH Trial [64], Now Everybody Together for Amazing and Healthful Kids (NET-Works) [65], Hip-Hop to Health Jr (Phase I) [61, 62] and the Ballabeina [66] study. Fifteen studies (39%) were found to be of moderate quality (50–79% of criterion), with three (8%) studies found to be of lower quality, meeting less than 50% of the criteria [67–71].

In terms of strengths, all studies addressed a clearly focused research question (100%), provided adequate details regarding randomisation (90%), included study groups similar at the start of the trial (84%), ensured that all study groups were treated equally (95%) and comprehensively reported the intervention effects (90%). However, notably, fewer studies accounted for all participants at its conclusion (63%), failed to adequately report the precision of the estimate of the intervention or treatment effect (63%) and/or ensured adequate blinding for either the participants, investigators, or assessors (40%) (Supplementary File S3).

### Characteristics of included studies

Most intervention studies were conducted in America (*n* = 35) [47–59, 61–65, 67–93], while the remaining three were conducted in Canada [94], the Netherlands [95] and Switzerland [66]. The sample sizes across the included studies ranged from 33 [89] to 4,999 [92] (*N* = 17,237), with an average sample size of 201 participants for the intervention conditions and 230 for the control conditions at baseline. Seven intervention studies (18%) were targeted at pregnant women [47, 48, 67, 72, 75, 80, 84, 94] or mothers with a newborn (< 14 weeks) [85] who were, on average, 27 years old at baseline. The remaining studies were targeted at children recruited from the age of 3–11 months [77, 90, 91] (*n* = 3), 12–23 months [78, 90, 96] (*n* = 3), 24–35 months [53–59, 61–63, 65, 68, 74, 76, 82, 86, 88, 93] (*n* = 13), 36–47 months [49–52, 64, 69–71, 73, 79, 81, 83, 89, 92, 95] (*n* = 11), and > 48 months [66] (*n* = 1), with an average age of 38 months at baseline. Around two-thirds of all intervention studies (*n* = 22) included a sample of a Hispanic and/or Latino majority [47–57, 63–65, 67, 72, 76, 77, 79, 83, 84, 86–89, 92–94], with a further seven and three including a black African American [57, 61, 62, 69–71, 73, 75, 78, 80, 81] and a Native American Indian [74, 85, 91] majority, respectively. Hip Hop to Health Jr (Phase I) in the first wave targeted a majority black African sample, with the second wave targeting a Latino majority [57–59]. The remaining five studies recruited a mixed sample with no clear majority [66, 68, 90, 95, 96] (see Table 2).

### Intervention characteristic

The average intervention length was 9.7 months (range 0.5–36), and the average length between the intervention finishing and the last follow-up measurement was 13.8 months (range 2.5–36). The behaviour targeted was physical activity in three intervention studies, diet in 12 studies, and diet and physical activity targeted across the remaining 23 studies [50–54, 56–59, 61–66, 68, 73, 74, 76, 78, 79, 82, 84, 86, 88, 90, 91, 93, 95].

Most interventions (*n* = 16) were delivered in a pre-school/childcare setting [51–54, 56–59, 61–63, 66, 68–71, 79, 83, 87, 89, 92, 95]. A further six were delivered in a medical setting, which included hospitals [78, 81] and outpatient/medical clinics [47, 48, 67, 77, 80], alongside six interventions delivered in the community, including recreation centres [49, 50, 76], community health centres [82, 86], places of worship [88], community fitness centres [90] and local parks [64]. Six studies were conducted solely in the home [74, 75, 84, 85, 91, 96], with a further three delivered in the family home alongside an additional setting such as childcare [73], hospital [94] and the community [65].

Most interventions targeted either the parents [47, 48, 65, 67, 72, 75, 77, 78, 80, 81, 84–86, 91, 92, 94] (*n* = 14) or the family [49, 50, 61, 62, 64, 66, 74, 76, 79, 82, 83, 87, 88, 90, 93, 96] (*n* = 13). The childcare/education provider and/or the child were targeted in four interventional studies [69–71, 89, 95], with the remaining seven targeted at parents, children and childcare/education providers [51–54, 56–59, 63, 68, 73]. The intervention provider across the studies was mixed, most commonly either a community worker/peer educator [49, 50, 54, 56–59, 63, 64, 74–78, 82–85, 88, 90, 91, 93, 94] (*n* = 19) or a preschool teacher/childcare provider [53, 61, 62, 66, 68–71, 73, 79, 89, 92, 95] (*n* = 10). Other intervention providers included registered dietitians [47, 48, 53, 72], medical practitioners/specialists [47, 48, 51, 67, 72, 96], psychologists [80], social workers [86], and graduate-level researchers [65, 81, 87]. Duration and frequency ranged from parents receiving a single 20-minute culturally tailored educational intervention on healthy beverage consumption [77] to parents receiving over 33 individual and group sessions delivered over 36 months [47, 48, 65, 72] (see Table 3).

None of the eligible studies provided explicit details on what behaviour change techniques (BCTs) were used within the interventions. Therefore, the CALO-RE taxonomy [25] was used to code eligible intervention studies with a range of 1–16 BCTs used within the studies. The number of BCTs used within the eligible interventions ranged from 1 to 15, with a mean of 6 (see S4 Table 1 for full BCT results). Identified BCTs were mapped onto the TDF; see S4 Table 2 for full TDF results.

With regard to the extent and type of cultural adaptation strategies used within each study, thirteen interventions (35%) exhibited comprehensive cultural adaptation. These studies demonstrated most or all five categories of cultural adaption [29]: peripheral, evidential, constituent involving, socio-cultural and linguistic [47, 48, 53, 54, 56–59, 63, 64, 72, 76, 83, 86, 88, 92, 93]. For example, The Coach Trial [64], developed using a community participatory approach, addressed barriers specific to that community, was delivered by Spanish-speaking health coaches and used foods relevant to the Latino community [57, 97]. Comprehensively adapted studies varied in the degree to which strategies were tailored at group, sub-group or individual level.

Ten interventions (26%) exhibited moderate cultural adaptation [61, 62, 68, 74, 77, 79, 80, 84, 85, 90, 91, 94]. Moderately ranked interventions typically employed at least two deep (e.g., socio-cultural and constituent involving) and one surface (e.g., peripheral) structure-based strategies. Often, these studies were limited in their linguistic adaptation compared to those rated as comprehensively adapted. For example, FunPals [90] had a good level of constituent involvement, utilising community stakeholders and parents as key informants to support the design and implementation of the curriculum and increase cultural relevance and appeal, but the study included no linguistic adaptation with fluent English stipulated within the participant inclusion criteria.

Fifteen studies (39%) exhibited minimal cultural adaptation [49, 50, 65–67, 69–71, 75, 78, 82, 87, 89, 96]. These studies tended to include fewer deep strategies (see Table 3). For example, within the ‘Mothers and Others’ intervention [75], whilst the study design was underpinned by a clear conceptual framework and expert resources were used to inform the anticipatory guidance curriculum, they do not report constituent-involving, socio-cultural or linguistic adaptation, despite this intervention’s focus on African American mothers and families.

### Primary outcome (arthrometric)

**Fig. 2:**
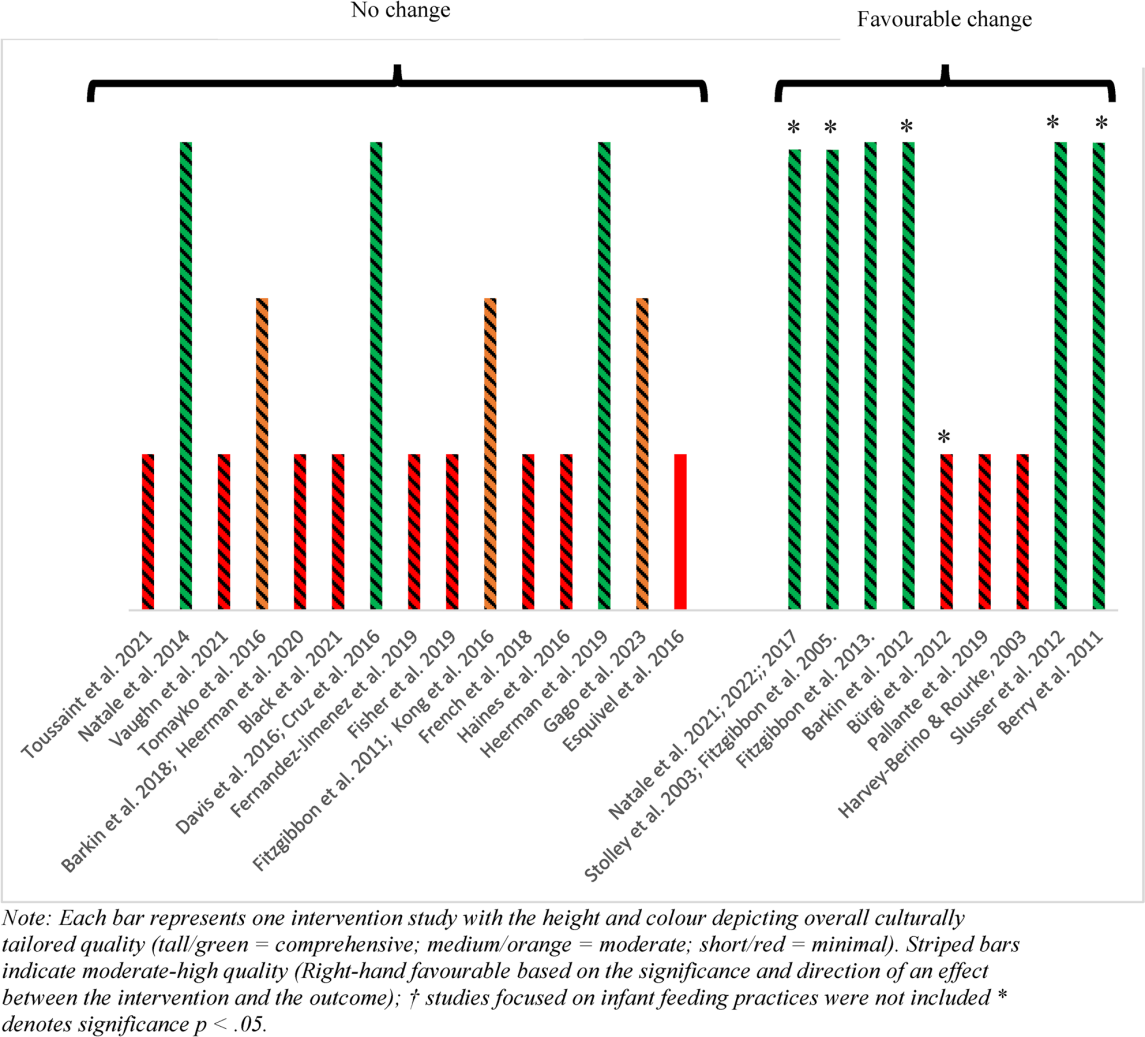
Anthropometric outcome/s

Twenty-four interventional studies^1^ reported changes in at least one anthropometric outcome. The effect size was calculated on the mean difference between control and intervention at post-intervention (or follow-up where provided) and used alongside *p* values to determine effects that favoured the intervention on the primary outcome. Most studies reported BMIz scores pre-and post-test, except for two intervention studies, which reported weight for age z score (WHz) [91] and body fat percentile [54, 55]. There was considerable heterogeneity related to the primary outcomes; for example, some papers reported a decrease in BMIz, with others reporting on whether there was a trajectory reduction in BMIz and/or body fat percentile, with others reporting if a healthy weight was maintained. Nine of the 24 (38%) interventions demonstrated an effect on an anthropometric outcome which favoured the intervention compared to the control [54–59, 63, 66, 76, 86–88, 91], five of which were significant [54–59, 66, 76, 86, 88] (see Fig. 2). Some papers conducted sub-group analysis, which identified larger interventional effects on children who were overweight/ obese at the beginning of the trial [76]. Further studies identified that preschool children in the intervention group revealed a slower linear BMI growth and/or reduced likelihood of being overweight or obese when compared to the control [49, 50, 83]. All except one intervention study that demonstrated a significant effect (83%) had a comprehensive cultural adaptation score [54–59, 76, 86, 88], and the majority of interventions that did not demonstrate an effect of the primary outcome which favoured the intervention were shown to be minimally culturally adapted (*n* = 9; 60%) (Fig. 2 and Table 4).

**Table 4:** Effect directions of primary and secondary outcomes

Study	Setting	Population targeted	BMI	BF	PA	Dietary intake	Parental feeding practices (infancy & early childhood)	Cultural adaptation	Quality
Alhassan et al. 2007	Pre-school	Children			↔			Minimal	Moderate
Anderson et al. 2005	Home and hospital based	Mothers		▲^*^				Moderate	High
Annesi et al. 2013	Pre-school	Children			▲^*^			Minimal	Low
Barkin et al. 2012	Community based	Parents and children	▲^*^					Comprehensive	Moderate
Barkin et al. 2018; Heerman et al. 2020	Community based	Parents and children	↔		↔	▲^*^		Minimal	High
Beck et al. 2017	Medical setting	Parents						Moderate	Moderate
Berry et al. 2011	Community based	Mothers/children	▲^*^					Comprehensive	Moderate
Black et al. 2021	Medical setting	Parents	↔		▲^2*^	↔^2^		Minimal	High
Bürgi et al. 2012	Pre-school	Parents, teachers, children	↔^2*^		▲^2*^			Minimal	High
Cepni et al. 2021	Community based	Parents and children			↔	↔^3^	↔^2^	Moderate	High
Davis et al. 2016; Cruz et al. 2016	Pre-school	Parents and children	↔		↔^2*^			Comprehensive	High
Esquivel et al. 2016	Pre-school	Parents, teachers, children	▼		▲^*^	↔^3^		Moderate	Low
Fernandez-Jimenez et al. 2019	Pre-school	Parents and children	↔					Moderate	Moderate
Fiks et al. 2017	Hospital	Mothers					▲*2	Moderate	High
Fisher et al. 2019	Mixed: Medical settings	Parents and children	↔			▲^3*^		Minimal	High
Fitzgibbon et al. 2002; Stolley et al. 2003; Fitzgibbon et al. 2005.	Pre-school	Parents and children	▲^*^		↔	▲^4*^		Comprehensive	High
Fitzgibbon et al. 2011; Kong et al. 2016	Pre-school	Parents and children	↔		↔	↔^4^		Moderate	High
Fitzgibbon et al. 2013.	Pre-school	Parents and children	▲		↔^3^	↔^4^		Comprehensive	Moderate
French et al. 2018	Home and community based	Parents and children	↔		↔	↔^4^		Minimal	High
Gago et al. 2023	Pre-school	Parents	▲^*^		↔	↔	↔	Comprehensive	Moderate
Gans et al. 2022	Pre-school	Parents and children			↔	▲^*^		Comprehensive	High
Haines et al. 2016	Community based	Parents and children	↔		↔	↔	↔^2^	Minimal	Moderate
Harvey-Berino & Rourke, 2003	Home based	Parents	▲		↔	▲^2^		Minimal	Moderate
Heerman et al. 2019	Community based	Parents and children	↔		↔	▲^3*^	▲^2*^	Comprehensive	High
Hughes et al. 2020	Pre-school	Parents and children					▲^6*^	Comprehensive	Moderate
Messito et al. 2020; Gross et al. 2016	Hospital/health centre	Mothers	↔^3*^	▲^2*^			▲^12*^	Comprehensive	High
Natale et al. 2014	Pre-school	Teachers and Parents	↔					Comprehensive	Moderate
Natale et al. 2017; 2021; 2022	Pre-school	Parents, teachers, children	▲^*^		▲^*^	↔^2^		Comprehensive	High
Nix et al. 2021	Home based	Parents and children				▲^*^	▲^2*^	Minimal	High
Pallante et al. 2019	Community based	Parents and children	▲		▲		▲	Minimal	Moderate
Petrova et al. 2009	Health Centre	Mothers		▲^*^				Minimal	Low
Reifsnider et al. 2018	Home based	Mothers	↔	↔				Moderate	Moderate
Rosenstock et al. 2021	Home based	Mothers	▲^3*^				▲^3*^	Moderate	High
Slusser et al. 2012	Mixed: Medical /community	Parents	▲^*^		▲			Comprehensive	High
Tomayko et al. 2016	Home based	Parents	↔		↔	↔		Moderate	High
Toussaint et al. 2021	Pre-school	Teachers	↔					Minimal	High
Vaughn et al. 2021	Pre-school	Parents and children	↔			↔	↔^4^	Minimal	Moderate
Wasser et al. 2020	Home based	Mothers	↔					Minimal	Moderate

Effect direction: upward arrow ▲= positive health impact, downward arrow ▼= negative health impact, sideways arrow ↔no change/mixed effects/conflicting findings. Subscript numbers: Number of outcomes within each category synthesis is 1 unless indicated in subscript beside effect direction, Smaller ▲ reflects low sample size (*N* < 100)

*BF B*reastfeeding,* PA *Physical activity

^*^p <. 05

**Fig. 3:**
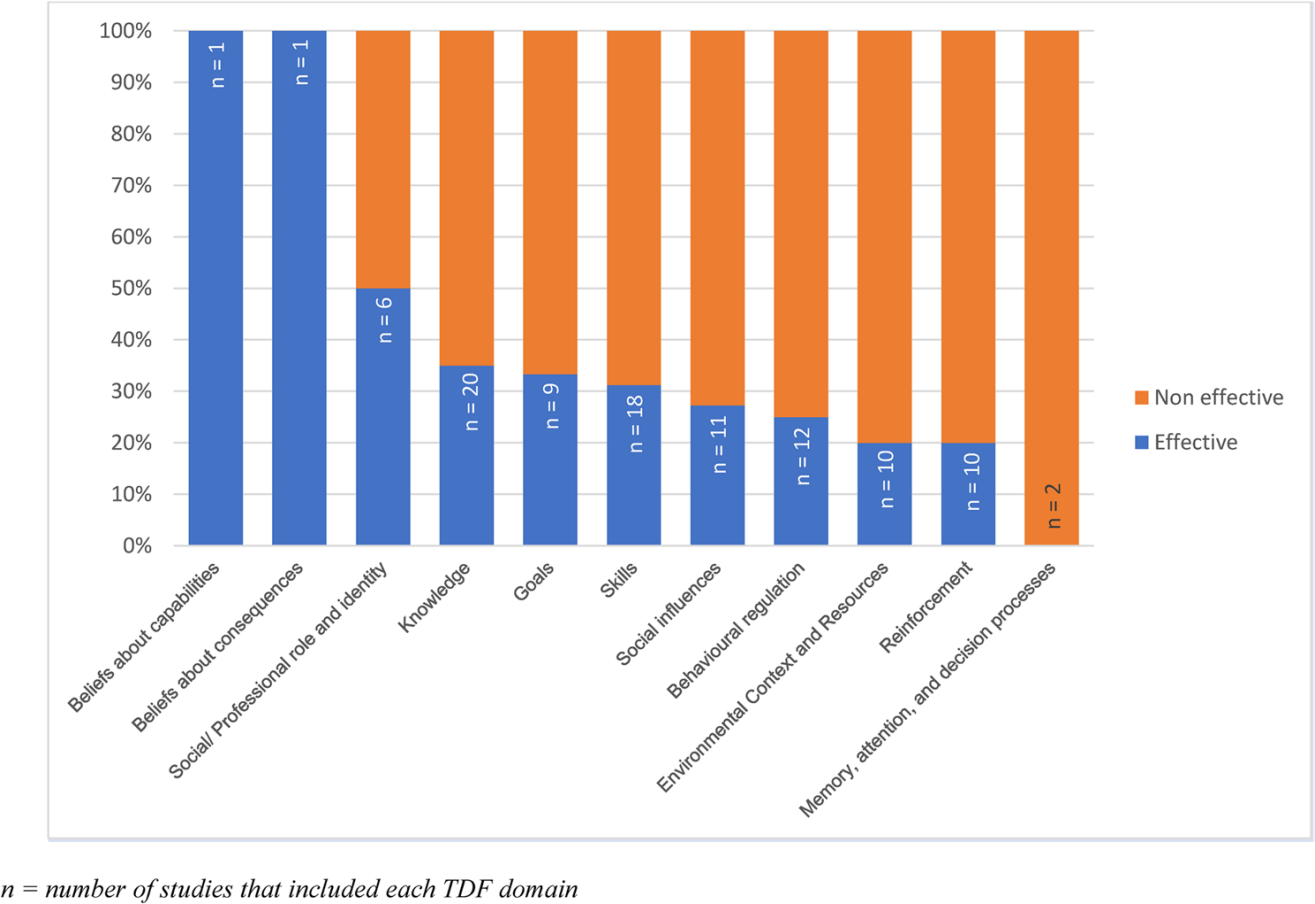
Percentage effectiveness ‘ratio’ of TDF domain targeted based on primary outcome

Eleven TDF domains were targeted across all intervention studies, including BMI Z score as an outcome. An effectiveness ratio was calculated to illustrate the relative weight of each TDF domain targeted; ‘*beliefs about capabilities’* and ‘*beliefs about consequences*’ had 100% effectiveness, and ‘*social/ professional role and identity’* had 50% effectiveness. The only non-effective domain targeted was ‘*memory*,* attention*,* and decision processes’* (Fig. 3). Across all interventions with an anthropometric outcome, 28 BCTs were identified as being used. One BCT had a 100% effectiveness ratio ‘*set graded tasks’* (effective in 1 out of 1 study), with five BCTs shown to have an effectiveness ratio of fifty per cent or higher *‘provide information on consequences of behaviour to the individual’* (66.67%; 2 out of 3), *‘model behaviour’* (54.55%; 6 out of 11), *‘provide rewards contingent on successful behaviour’* (50%; 2 out of 4), *‘prompt self-monitoring of behaviour’* (50%; 3 out of 6) and *‘stress management’* (50%; 2 out of 4) *(*see Supplementary file S5 Fig. 1).

### Secondary outcome/s

Given the extent and variation of secondary outcomes reported across the studies, the outcomes most widely reported were synthesised; dietary intake (*n* = 17), physical activity (*n* = 24), and parental feeding practices (*n* = 18; during infancy *n* = 7 during early childhood *n* = 9). Child behavioural outcomes related to sedentary behaviour, screen time and sleep and parental outcomes related to physical activity and/or media-related parenting practices and attitudes were not included in the evidence synthesis.

#### Child behavioural outcomes

##### Dietary intake

Eighteen interventional studies reported on dietary intake, which was assessed using self-reported fruit and vegetable intake (*n* = 8), Healthy Eating Index (HEI) [98] (*n* = 5), calorie/fat intake (*n* = 3) or healthy eating habits (% meals/snacks over 3 days that included a fruit and/or vegetable, a source of protein, and no sweets or junk food) [96] (*n* = 1). Seven interventional studies were shown to have effects which favoured the intervention, six of which were significant when compared to the control [57–59, 64, 76, 81, 93, 96]. Three interventions (42%) that found a significant favourable change compared to the control on dietary intake were comprehensively culturally tailored [57–59, 64, 93]. The remaining 11 studies found inconclusive and/or mixed findings, eight of which were minimally to moderately culturally adapted (73%) (Table 4). 

**Fig. 4:**
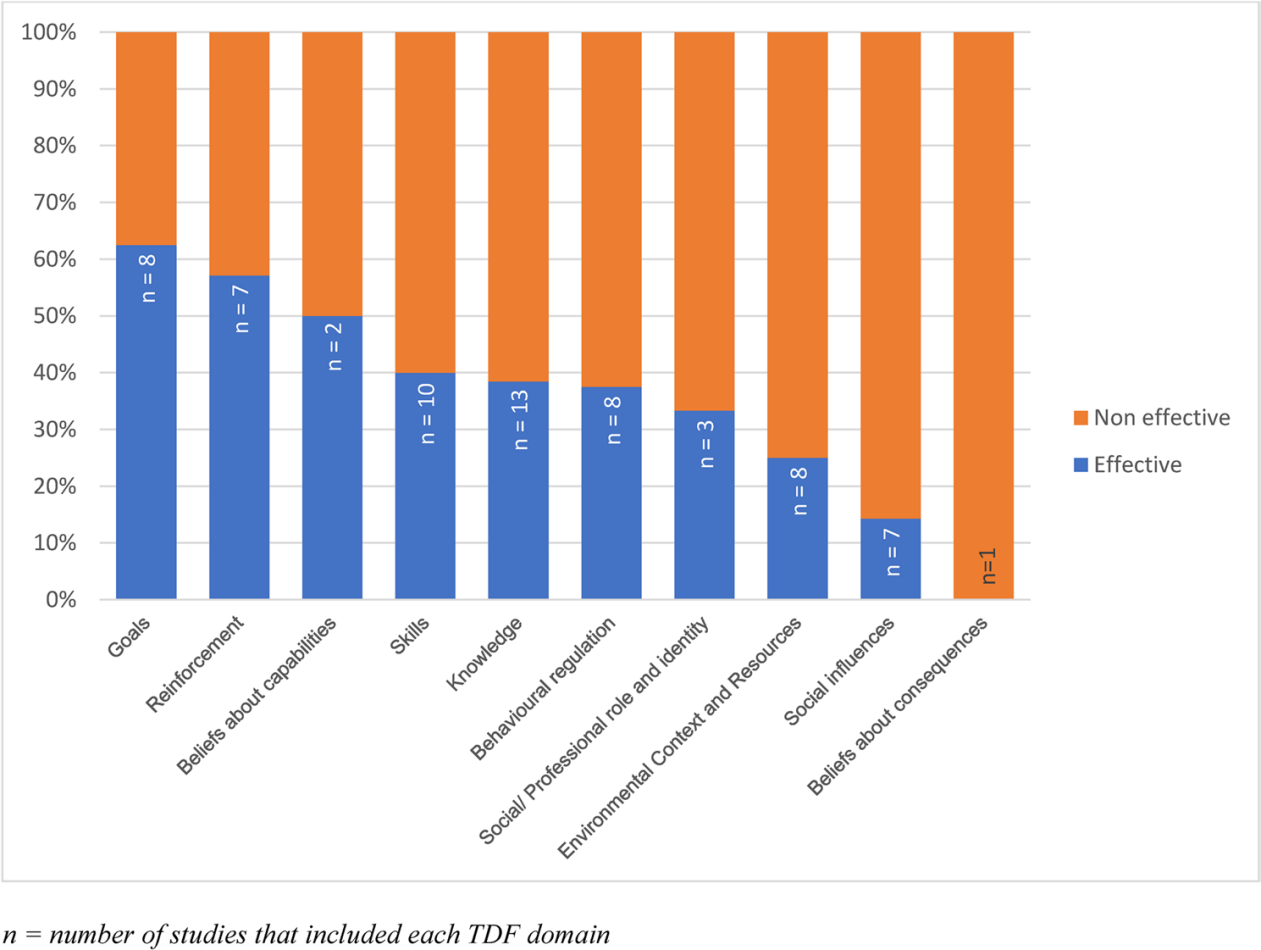
Percentage effectiveness ‘ratio’ of TDF domain targeted based on dietary intake outcomes

Ten theoretical domains were shown to be targeted across the intervention studies that had a dietary intake outcome. The domains ‘*goals’* (62.5%), ‘*reinforcement’* (57.1%) and ‘*beliefs about capabilities’* (50%) achieved the highest effectiveness ratio, with ‘*beliefs about consequences*’ shown to be the only TDF domain that was not associated with effectiveness (Fig. 4). A total of 36 BCTs were used, with two only found in effective trials; ‘*provide information on consequences of behaviour to the individual’ and* ‘*goal Setting (outcome)*’. BCTs ‘*barrier Identification’* (83%; 5 out of 6), ‘*prompt rewards contingent on effort or progress towards behaviour’* (75%; 3 out of 4), ‘*environmental restructuring’* (75%; 3 out of 4), ‘*prompt identification as a role model’* (75%; 3 out of 4), ‘*provide rewards contingent on successful behaviour’* (67%; 2 out of 3), ‘*motivational interviewing’* (67%; 2 out of 3), ‘*prompt review of behavioural goals’* (50%; 2 out of 4) and ‘*stress management’* (50%; 2 out of 4) were also shown to score highly (see Supplementary file S5 Fig. 2). There were several BCTs which were used in more than one study but were not shown to be effective, including; *‘prompt review of outcome goals’* (0 out of 4), ‘*shaping’* (0 out of 5), *‘prompt generalisation of a target behaviour’* (0 out of 5), ‘*prompt focus on past success’* (0 out of 7), *‘provide information on where and when to perform behaviour’* (0 out of 3), ‘*teach to use prompts’* (0 out of 10), ‘*agree to behavioural contract’* (0 out of 3) and ‘*prompt anticipated regret’* (0 out of 2) (see Supplementary file S5 Fig. 2).

##### Physical activity

**Fig. 5:**
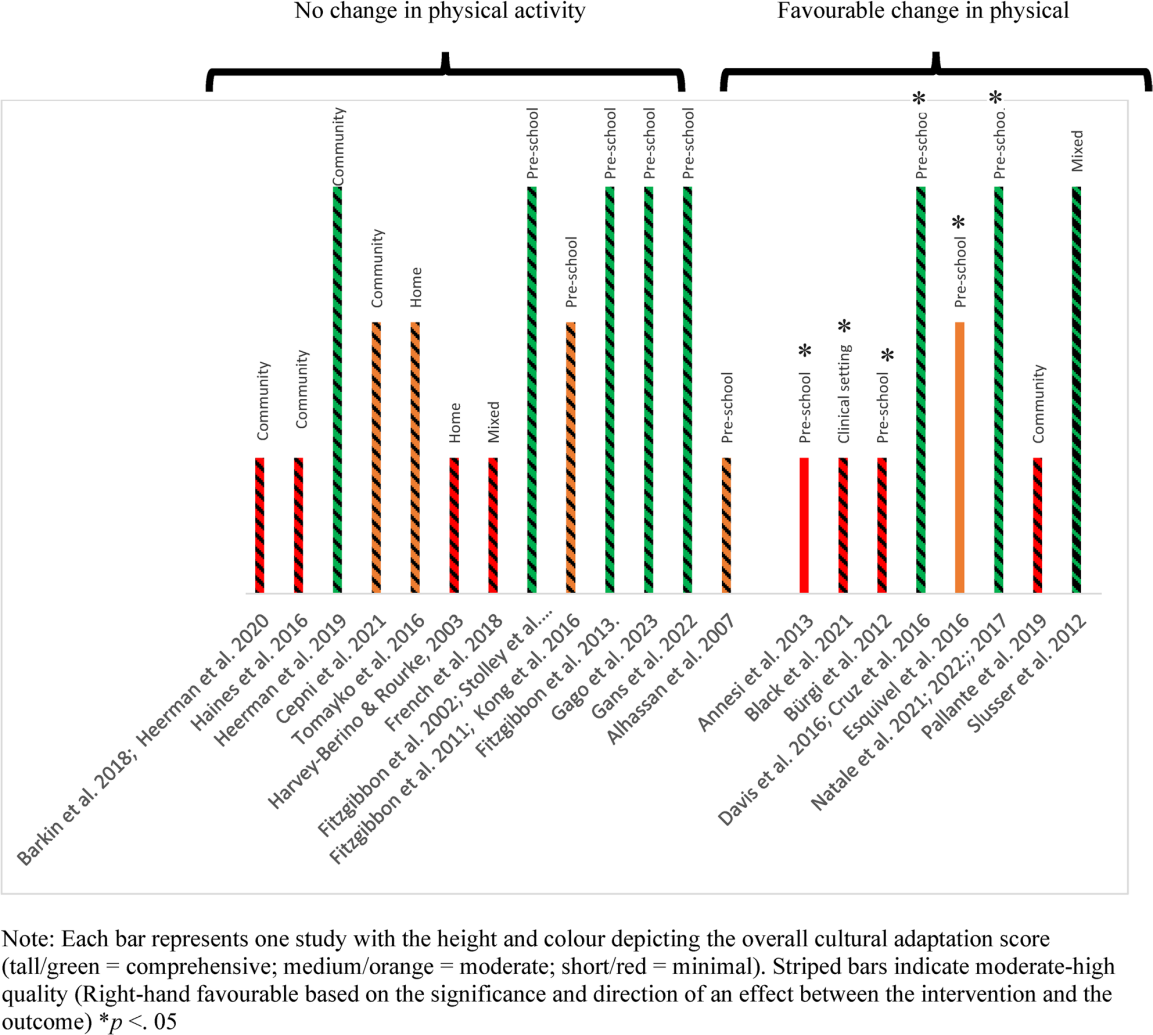
Intervention studies demonstrating favourable effects on physical activity compared to the control group

Twenty-one interventional studies reported physical activity outcomes pre- and post-intervention. Eight of these studies demonstrated a direction of the effect favouring the intervention, six of which were significant. Thirteen studies showed no effect and/or mixed findings (Fig. 5). The most commonly used outcomes for physical activity reported were moderate-vigorous physical activity (MVPA) (*n* = 6) [49, 50, 65, 69–71, 90, 93], parent self-report (*n* = 2) [86, 87], physical activity monitors (*n* = 3) [74, 89, 91], the Environment and Policy Assessment and Observation (EPAO) (*n* = 2) [54–56, 68]. Three of the eight interventions that demonstrated a direction of effect which favoured the intervention had a comprehensive cultural adaptation score [51, 52, 54–56]. Six interventions demonstrated a significantly favourable outcome; two (33%) had a comprehensive cultural adaptation score, and five (83%) were delivered in the preschool setting. None of the interventions delivered at home were found to have effects that favoured the intervention on physical activity (Table 4).

**Fig. 6:**
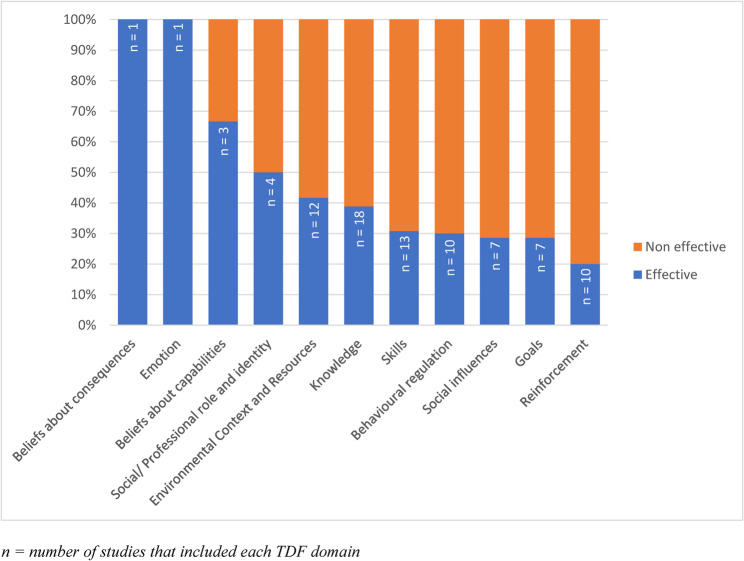
Percentage effectiveness ‘ratio’ of TDF domain targeted based on physical activity outcomes

Ten TDF domains were targeted with ‘*emotion*’ and ‘*beliefs about consequences*’, both achieving a 100% effectiveness ratio and ‘beliefs about capabilities’ (67%; 2 out of 3), ‘social/ professional role and identity’ (50%; 2 out of 4) and ‘environment context and resources’ (42%; 5 out of 12) also shown to score highly (Fig. 6). A total of 26 BCTs were used, of which eleven had a 100% effectiveness ratio; ‘*provide normative information about others behaviour*’, ‘*goal Setting*’, ‘*set graded tasks*’, ‘*prompt review of behavioural goals*’, ‘*prompt review of outcome goals*’, ‘*provide feedback on performance*’, ‘*teach to use prompts*’, ‘*agree to behavioural contract*’, ‘*facilitate social comparison*’, ‘*prompt self-talk*’ and ‘*stress management*’. However, caution should be taken as these BCTs were only used in one study (100% 1 out 1). BCTs ‘*provide instruction on how to perform the behaviour*’ (71%; 5 out of 7), ‘*provide information on consequences of behaviour to the individual*’ (67%; 2 out of 3), ‘*provide rewards contingent on successful behaviour*’ (50%; 2 out of 4), ‘*prompt self-monitoring of behaviour*’ (50%; 2 out of 4), ‘*plan social support*’ (50%; 2 out of 4) and ‘*prompt identification as a role model*’ (50%; 2 out of 4) were also shown to score highly (see Supplementary file S5 Figure 3).

#### Parental outcomes: parental feeding practices

Studies reporting parental feeding practices in infancy were synthesised separately from those that reported on feeding practices during early childhood.

##### Infant feeding practices

Seven intervention studies reported outcomes relating to parental feeding practices in infancy [47, 48, 67, 72, 75, 80, 84, 85, 94], four of which reported breastfeeding outcomes (e.g. duration of exclusive breastfeeding) [47, 48, 67, 72, 84, 94]. Additional infant feeding outcomes explored across the studies were adding cereals to bottles (*n* = 1) [80], age of introduction to solid foods (*n* = 1) [85], infant consumption of sugar and sweetened beverages (*n* = 1) [85], responsive feeding (*n* = 3) [47, 48, 72, 80, 85] and controlling feeding (e.g. pressure) [47, 48, 72, 77]. Three interventions reported a significant and favourable increase in the duration of exclusive breastfeeding [47, 48, 67, 72, 94], one a reduction in adding cereals to bottles [80], one an increase in the age of introduction to first foods [85], one with a reduction in infant consumption of sugar and sweetened beverages (*n* = 1) [85], three ran increase in responsive feeding [47, 48, 72, 80, 85] and two a significant reduction in pressure to eat [47, 48, 72, 80]. The majority of interventions were delivered by bilingual peer educators (*n* = 5), were individual (*n* = 4) [75, 84, 85, 94] or a mixture of individual/group sessions (*n* = 4) [47, 48, 67, 72, 80] delivered across a median of 8 sessions (2 to 28) and a duration of 11 months (3 to 36 months). Four of the five interventions, which were shown to reveal a direction of effect that favoured the intervention on feeding practices during infancy [47, 48, 67, 72, 80, 85, 94], were either moderately [80, 85, 94] or comprehensively [47, 48, 72] culturally tailored. Two of these interventions were also shown to have a favourable effect on longer-term weight outcomes [47, 48, 72, 85] compared to the control. However, the positive effect for one of these studies was not sustained [47, 48, 72] (Table 4).

**Fig. 7:**
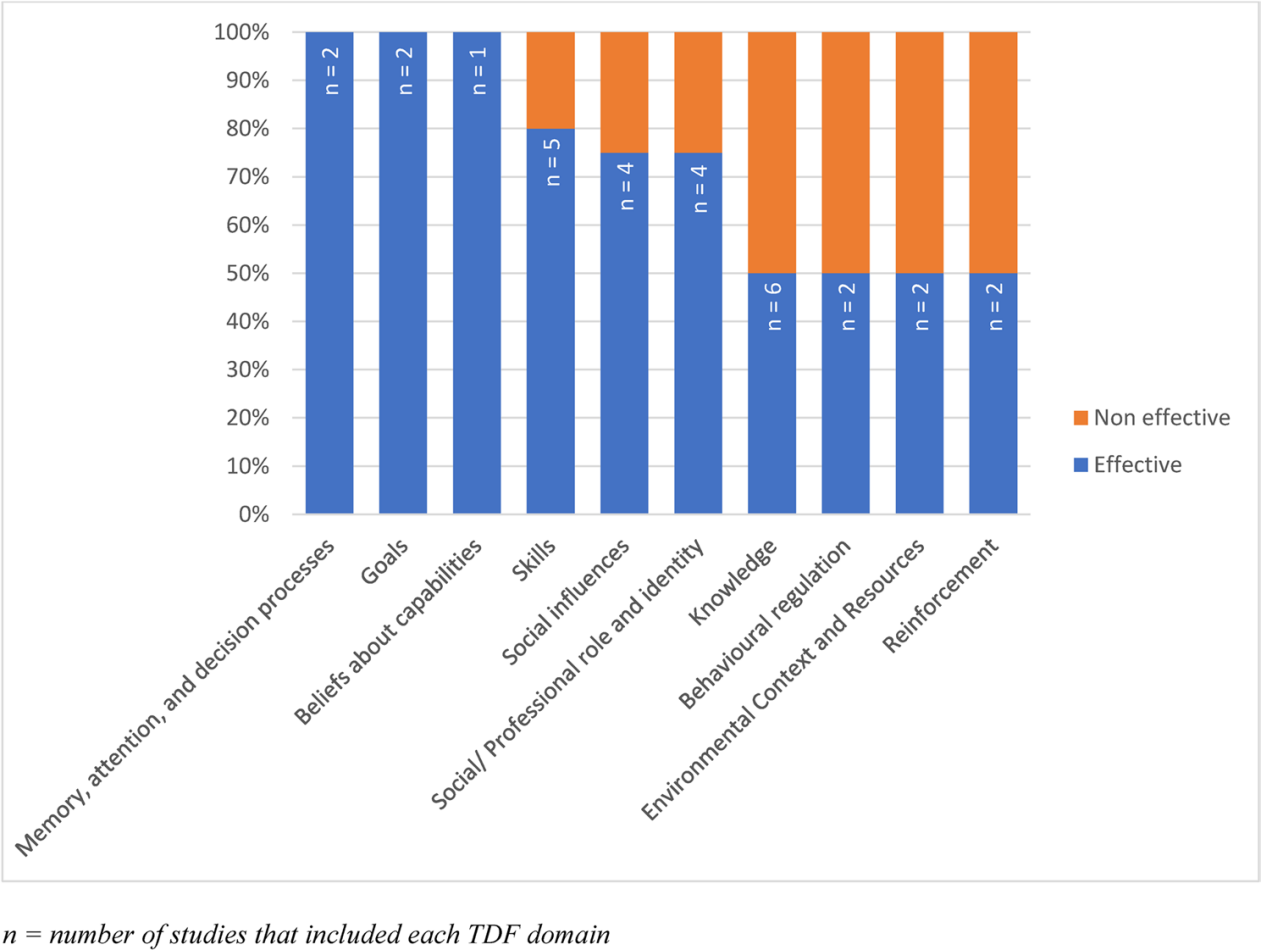
Percentage effectiveness ‘ratio’ of TDF domain targeted in infant feeding interventions

Eight theoretical domains were identified to be targeted across all interventions that targeted infant feeding practices, with four domains, ‘*optimism*’, ‘*beliefs about consequences*’, ‘*intentions*’, and ‘*emotion*’ not targeted across any studies. All of the TDF domains targeted were shown to have at least a 50% effectiveness ratio or more in relation to infant feeding practices, with ‘*goals*’ (100%), ‘*memory*,* attention*,and * decision processes*’ (100%), ‘*skills*’ (80%), ‘*social influences*’ (75%) and ‘*social/ professional role and identity*’ (75%) shown to be associated with the most effective interventions (Fig. 7). A total of 17 BCTs were used across all the studies that targeted infant feeding practices. There were eight BCTs which achieved 100% effectiveness, which included ‘*provide feedback on performance*’ *(effective in 3 out of 3 studies)*, ‘*model behaviour*’ (*n* = 3), ‘*goal setting (behaviour)* (*n* = 2), ‘*action planning*’ (*n* = 1), ‘*prompt focus on past success*’ (*n* = 1), ‘*facilitate social comparison*’ (*n* = 1) ‘*environment restructuring*’ (*n* = 1) and ‘*motivational interviewing*’ (*n* = 1). The BCTs ‘*plan social support/social change*’ (75%, effective in 3 out of 4 studies) and ‘*prompt identification as a role model*’ (75%; 3 out of 4) also scored highly (see Supplementary file S5 Fig. 4).

##### Parental feeding practices and attitudes in early childhood

Seven interventional studies [64, 73, 82, 83, 90, 92, 96] reported on parental feeding practices and attitudes in early childhood. There was large heterogeneity in outcomes reported and measurement tools used, with papers reporting on a range of feeding practices (e.g. pressure to eat, restriction, modelling, encouragement, portioning, involvement and responsive feeding), the feeding environment (e.g. availability of food, and family meal patterns and routines) and parental attitudes (e.g. empowerment and feeding efficacy).

Significant favourable effects were shown in four interventions [64, 82, 83, 96] with an increase in responsive feeding (*n* = 2) [83, 96], parental feeding self-efficacy (*n* = 2) [64], offering new foods [83], measuring portions [83] and involving children in food preparation [83], and a decrease in restriction of food intake [82]. However, results were mixed [82, 96], and only two of these studies reported significant favourable effects in multiple parental feeding practices [82, 83], both of which had a comprehensive cultural adaptation score. Of the five studies reporting mixed findings or no effects [73, 82, 90, 92, 96] four were shown to have a minimal to moderate cultural adaptation (*n* = 4; 80%) (Table 4).

## Discussion

This systematic review is the first to synthesise and determine which behaviour change techniques and cultural adaptation strategies have been used in interventions targeting childhood obesity in young minority ethnic children (aged < 5 years). This review included 38 unique intervention studies (published across 49 papers). The studies we identified targeted a range of outcomes and behaviours, with improvements noted for weight change, infant feeding practices, reduction in dietary fat intake and increased physical activity. The studies included were all randomised controlled trials with various methodological quality ranging from low to high. The majority of studies appraised were identified to be moderate or high quality, with lower quality studies determined to be related to lack of accounting for participants at the conclusion, failure to report the precision of the treatment effect and/or ensuring adequate blinding; the findings from these studies should be interpreted cautiously.

Around one-third of interventional studies (*n* = 9) demonstrated a favourable difference in the primary outcome compared to the control, which was significant in five studies. The majority of studies that revealed a significant difference (*n* = 4; 83%) were shown to be comprehensively culturally adapted and used a range of deep and surface adaptation strategies. In contrast, minimally ranked interventions showed minimal effectiveness in relation to the primary outcome. Similar findings were also found for dietary change and parental feeding practices during early childhood, whereby minimally ranked interventions were associated with minimal or mixed effectiveness, with most interventions ranked either moderately or comprehensively culturally adapted, associated with positive effects on parental feeding practices. Whilst these findings cannot provide a causal inference that cultural adaptation impacted the efficacy of the interventions, it does add weight to the growing body of evidence that emphasises the value and importance of culturally competent interventions when engaging with diverse populations to improve health outcomes and reduce inequalities [27], which inform practical suggestions for adaptation strategies that may enhance health promotion interventions. Things to consider include ensuring the audio or visual elements of an intervention reflect the lived experiences of the ethnic group (e.g. music, pictures, foods, people), utilising participatory approaches to incorporate input from the target community/stakeholders and imbed social and cultural values into the intervention design and linguistic strategies, with bilingual facilitators and culturally equivalent translations of materials and measurement instruments.

The scoring system used for this study [27] also considered ‘tailoring’, which refers to the extent to which an intervention is or can be modified to reach a subgroup or individual [99, 100]; an important consideration given the wide variability not just between but within and among cultural groups [101]. However, whilst the interventions rated as ‘comprehensive’ within this review adopted a range of surface and deep adaptation strategies, few were tailored to sub-group or individual level. Castro, Barrera [102] discuss the tension dynamic between the fidelity of implementation, delivering the intervention as prescribed, and program adaptation, modification or tailoring towards a specific group, sub-group or individual. Further research is needed to unpick the extent to which individual or sub-group tailoring is required and its impact on both fidelity and intervention effectiveness.

Most interventions that demonstrated a favourable outcome for child weight or dietary change were delivered in the community and targeted at children alongside their primary caregivers. Whilst fourteen interventional studies were delivered in preschool settings, only one of these demonstrated a favourable effect. Education settings, such as pre-schools and schools, often represent a popular setting for implementing upstream public health interventions [103], providing a cost-effective strategy that can reach multiple target groups and providers [104] with the necessary infrastructure to facilitate implementation [105]. Whilst this may be the case, the findings from this review suggest that the sole reliance on using schools as a context to engage with diverse families may not be sufficient to impact change in weight status and/or childhood nutrition practices. This finding highlights the importance of considering the setting when delivering interventions to diverse communities and utilising multiple settings (home and school) when targeting children’s weight-related behaviours [106]. This finding may reflect the challenges that teachers and other educational stakeholders face in initiating conversations concerning children’s weight-related behaviour change alongside a lack of collaboration between teachers and parents [107]. Increasing research capacity through collaboration and partnerships with minority ethnic families alongside wider community stakeholders using community-led system approaches may offer a more effective solution to ensure obesity prevention interventions remain tailored to the local context, which will provide sustainable solutions that can deliver increased impact [108].

Developing an effective intervention is contingent on having a comprehensive understanding of the target behaviours and the determining factors that influence the target behaviour [41]. The underpinning of theory is an integral aspect in the intervention development process, providing a framework to determine how change will occur, increasing understanding of how the intervention intends to work, and ensuring that the specific intervention techniques used within the intervention remain driven by this theory to bring about change [42, 109]. However, the reporting of the underpinning behaviour change theory that guides an intervention is often underreported or implicit [110, 111], as was the case in many of the included studies [66, 94, 112]. Whilst this review did not identify the theoretical underpinning of the included studies, through mapping the interventions to the Theoretical Domains Framework (TDF) [42], it has provided an increased understanding of the mechanisms that may influence intervention success. This review revealed that interventions targeting the theoretical domains of knowledge, skills, social, professional role and identity, behavioural regulation, social influences and goals revealed the most promising changes concerning child weight and/or dietary change. In support of previous research [21, 24], this review identified that the intervention techniques associated with the most promising success related to goal setting, barrier identification, role modelling and increasing self-efficacy through self-regulation and prompting practice of behaviour and provide useful behaviour change techniques for future interventions that seek to impact child weight and/or dietary change.

The findings confirmed that nineteen of the included intervention studies reported on physical activity outcomes, with the majority (89%) identified as multi-component interventions targeting more than one behavioural outcome. Eight of these intervention studies targeting physical activity outcomes appeared less important, with only two interventions demonstrating significant effects that were comprehensively tailored. The cultural adaptation of interventions targeting physical activity outcomes appeared less important, with only two interventions demonstrating significant effects that were comprehensively tailored. However, most studies that reported significant effects in physical activity outcomes were related to policy-level interventions delivered in a preschool setting that targeted children, parents, and teachers. Further, intervention studies, which targeted the theoretical domains of knowledge, environmental context, resources, skills, and behavioural regulation, revealed the most promising changes in physical activity. Therefore, this suggests, in line with previous research [110], that educational settings may have a more promising role in changing physical activity behaviour than dietary change in young minority ethnic children.

Early childhood interventions were found to provide promise, with five out of seven (71%) shown to have a favourable effect on breastfeeding and/or infant feeding practices, two of which revealed a positive impact on weight outcomes. Three out of four (75%) interventions that reported breastfeeding outcomes revealed a significant improvement in exclusive breastfeeding rates. Most successful infant feeding interventions were either moderately or comprehensively tailored and were often delivered through peer bilingual educators. This review revealed that interventions targeting the theoretical domains of skills and memory, attention and decision processes (psychological capability), social influences (social opportunity) and social, professional role and identity, and goals (reflective motivation) revealed the most promising changes related to infant feeding practices. When mapped to the Behaviour Change Wheel (BCW) [41], intervention functions such as education, training, enablement, and modelling may provide useful intervention approaches to influence positive change in infant feeding practices [41].

### Strengths and limitations

This is the first review to synthesise the evidence to examine the effects of behavioural interventions to prevent childhood obesity in minority ethnic children. This review uniquely brings together evidence of the cultural adaptations used, and theoretical domains targeted, alongside the behaviour change techniques used based on the CALO-RE taxonomy [25], to better understand both the theoretical and contextual factors that may be associated with the intervention effects. This evidence provides a more comprehensive understanding of which specific intervention components are most effective in reducing obesity in high-risk populations, which is of critical importance to decision-makers [113]. The strengths of this review include the comprehensive terms and databases searched, the RCT design of the studies included, and the pre-registration of the protocol, with all titles and abstracts double-screened to add additional rigour.

There are some limitations which are noteworthy for discussion. Due to poor reporting of ethnicity in some of the primary studies retrieved, some potentially relevant studies may have been excluded, as it was not possible to confirm the ethnic backgrounds of the participants. The BCTs, TDF, and cultural adaptation coding were based on what was reported in the manuscript. Inconsistent reporting and a lack of detail in this regard may have resulted in inaccurate scoring. Whilst the introduction of the Template for Intervention Description and Replication (TIDieR) has made great efforts to improve the quality of reporting interventions [114], there is a need to better capture the role of context and adaptations of intervention delivery [115], particularly important for interventions that are culturally tailored and/or delivered across diverse settings/stakeholders. This would enhance the interpretability and reproducibility of findings, thereby enabling future implementers to critically assess the applicability of identified factors within their specific contexts and to anticipate necessary adaptations or contextual modifications [115]. In addition, the cultural adaptation scoring [27] used within this study places a substantial emphasis on linguistic strategies, therefore limiting the adaptation score of those studies that did not adopt these. Whilst this may be an important consideration for some minority ethnic groups, it may be less relevant for other subcultural populations who are highly acculturated [102]. Therefore, future research should further explore which elements of cultural adaptation may be most important and within what context. Finally, study samples differed in the degree to which children were of healthy weight/overweight at baseline, which, in addition to heterogeneity in measurement, analysis and reporting of anthropometric data, limited the interpretation and synthesis of data relating to the primary outcome. Some studies identified larger interventional effects for children who were overweight/obese at the beginning of the trial [76], suggesting that sub-group analyses may be prudent in future RCTs exploring the impact of interventions on early growth patterns within community samples. Longer-term follow-ups are needed to explore sustainability and long-term impact.

## Conclusion

Our review has uncovered the importance of cultural adaptation within interventions that aim to facilitate change in obesity-preventative practices, particularly those related to infant feeding, dietary intake and parental feeding practices. It has provided further evidence that linguistic, constituent-involving and socio-cultural strategies that prioritise community and stakeholder participation and embed social and cultural values into the intervention design may improve program effectiveness [116, 117]. However, whilst all interventions included in this review targeted minority ethnic children, only one-third of these demonstrated comprehensive cultural adaptation [29]. These findings highlight the need for a greater emphasis on cultural adaptation to ensure that interventions have increased relevance and impact [27]. However, given the wide variability that exists not only between but also within cultural groups [100], few studies considered the modification or tailoring towards a specific group, sub-group or individual. Therefore, further research is needed to unpick the extent to which individual or sub-group tailoring is required and its impact on both fidelity and intervention effectiveness.

Early childhood represents a critical period of development that predicts growth patterns and associated health outcomes into adulthood [118]. Yet despite the high prevalence of childhood obesity reported among minority ethnic populations in the UK [4–6], most trials identified for review were based in America, with none in the UK. Further research should seek to review and, if necessary, refine the scoring system used in this review, incorporating input from relevant stakeholders, to establish a validated framework for assessing cultural adaptation of behavioural interventions within the UK context. This tool will be essential for intervention development, ensuring the relevance, acceptability, and effectiveness of interventions that can better meet the needs and values of diverse populations in the UK. Furthermore, there remains an urgent need for high-quality randomised controlled trials to assess the efficacy and cost-effectiveness of evidence-based obesity prevention interventions in early childhood, particularly those designed specifically for ethnically diverse populations in the UK. Financial investment is therefore required through research funding agencies to prioritise funding to generate a strong evidence base of the most effective upstream interventions tailored for minority ethnic families. This evidence will seek to reduce the equity gradient, which remains critical to decision-makers and policymakers [113].

## Supplementary Information

Supplementary Material 1.

Supplementary Material 2.

Supplementary Material 3.

Supplementary Material 4.

Supplementary Material 5.

## Data Availability

The research protocol was registered with PROSPERO and can be found under registration number CRD42022348116. The search strategies can be found in the appendices. The datasets used and/or analysed during the current study are available from the corresponding author upon reasonable request.

## Footnotes

1 Excluding interventions focused on infant feeding practices.
