# Supplementary material for: Effects of behavioural interventions for preventing obesity in young children from ethnic minority backgrounds: a systematic review of randomised controlled trials
Source: Arch Public Health. 2026 May 18;84:153. doi: 10.1186/s13690-026-01951-x (PMC13352745; doi:10.1186/s13690-026-01951-x)
Supplement: Supplementary file 2 — Supplementary Material 2. [file 13690_2026_1951_MOESM2_ESM.docx]

# Supplementary File 2

#### Table 1: Search strategy Ebsco Medline, CINAHL, PsycINFO <1995 to 1^st^ November 2024>

| **Number** | **Concepts** | **Key words and combinations** |
| --- | --- | --- |
| 1 | **Population**  Ethnicity related  Combined with OR | MeSH terms: ethnic and racial minorities, minority groups, emigrants and immigrants, cultural diversity, ethnicity [EXP], refugee*, African Americans, Arabs, Asian American, Indigenous Peoples, Jews, Roma, Blacks [EXP], American Native Continental Ancestry Group [EXP], Asians [EXP], Native Hawaiian, other Pacific Islander, Continental Population Groups [EXP] |
|  |  | Free text terms (ab.ti.): ethnic*, ethnic* group, minorit*, “ethnic* minorit*, minorit* group, rac* minorit*, foreign*, cultur*, diversit* B*ME, black Asian Minority Ethnic, multiethnic*, multi* ethnic*, mixed ethnic*, multi rac*, multi*cultural, multicultural, cross cultural, transcultural, emigra*, immigra*, migra*, asylum seeker, indigenous, refugee, transient, black, Afr*, Caribbean, Asian, Pakistani, Bangladesh*, India*, Chin*, Sri Lanka*, South Asia, Hispanic, Latin*, Iraqi, Arab, Aboriginal*, Traveller*, Gyps*, Roma |
| 2 | **Population**  Children (aged 0-5) related  Combined with OR | MeSH terms: child [EXP], infant [EXP], nurseries, infant, minors |
|  |  | Free text terms (ab.ti.): child*, infan*, young, paed*, kid, bab*, pediat*, preschool, toddler* |
| 3 | **Intervention**  Intervention/Lifestyle modification related  Combined with OR | MeSH terms: health promotion [EXP], social support [EXP], family therapy [EXP], health education (EXP], intervention studies, behaviour therapy |
|  |  | Free text terms (ab.ti.kw):: primary prevention, secondary prevention, preventative measure*, prevent*, behav* chang*, behave* intervention, behav* program, behavio*r chang*, behavio*r interv*, behavio*r program, “behav* modification, behavio*r modification, promotion of health, promotional items, program*, promot*, health promot*, health Campaign*, campaign, soc* interv*, lifestyle interv*, lifestyle chang*, lifestyle intervention, life style chang*, behav* change techn*, soc* support, peer support, couns*, behav* couns*, lifestyle couns*, couns* educ*, diet advice, diet educ*, health educ*, behav* educ*, psych* interv*, lifestyle change, life style change, commun* interv*, family interv*, parent* interv*, lifestyle mod*, obesity prevent*, physical educat* |
| 4 | Feeding/Physical Activity related  Combined with OR | MeSH terms: exercise [EXP], breast Feeding [EXP], bottle feeding, food preferences, diet, healthy, food fussiness |
|  |  | Free text terms (ab.ti.): exercis*, activ*, aerobic exercis*, leisure activ*, exercis* therapy, physical Fitness, group exercis*, physical activ*, activity change, walk*, run*, jog*, danc*, skip*, swim*, outdoor activit*, outdoor game*, game*, play*, play time, play* games, breastmilk, breastfed, breastfeed*, breast-feed*, breast-fed, Infant feed*, human milk, bottle fe*, infant formula feeding, “cow* milk, fortify, wet nursing, milk sharing, mealtime*, feed*, feeding beh*, feeding pattern*, feeding practice*, food*, eat*, diet*, eating beh*, eating practic*, diet* intake, diet* habit*, food habit*, eating habit*, nutrit*, energy intake, food intake, macronutrient*, micronutrient*, health* nutrit*, wean*, complim* feed*, solid feed* |
| 5 |  | #1 AND #2 AND #3 AND #4 |
| 6 |  | #1 AND #2 AND #3 AND #4 with a search limit date of date of publication: 01/01/1995-Current and narrowed by subject age: infant, newborn: birth-1 month; infant: 1-23 months; all infant: birth-23 months; child, preschool: 2-5 years. |

NB: Search terms were combined with ‘OR’ and concepts were combined with ‘AND’

#### Table 2: Search strategy SCOPUS <1995 to June Week 4 2023>

| **Number** | **Concepts** | **Key words and combinations** |
| --- | --- | --- |
| 1 | **Population**  Ethnicity related | ( TITLE-ABS-KEY ( "ethnic and Racial Minorities" OR "minority Groups" OR "emigrants and immigrants" OR "cultural diversity" OR "ethnicity" OR refugee* OR "african americans" OR "Arabs" OR "Asian American*" OR "Indigenous Peoples" OR jews OR roma OR "Blacks" OR "American Native Continental Ancestry Group" OR "Asians" OR "Native Hawaiian or Other Pacific Islander"  OR  "Continental Population Groups" OR ethnic* OR ethnicity OR "ethnic Group" OR minorit* OR "ethnic minorit*" OR "Minorit* group" OR "Racial minorit*" OR foreign* OR rac* OR  racial* OR cultur* OR diversit* OR  b*me OR "Black Asian OR minority ethnic" OR multiethnic OR "multi* ethnic*" OR "mixed ethnic*" OR "multi rac*" OR "multi*cultural" OR "multi cultural" OR "cross*cultural" OR "cross cultural” OR "trans*cultural*" OR "transcultural"  OR  emigra* OR immigra* OR migra* OR "asylum seeker" OR indigenous OR refugee OR transient OR  black  OR  afr* OR Caribbean OR asian OR Pakistani OR bangladesh* OR  india* OR china OR chinese OR  "sri lanka*" OR "south Asia" OR hispanic OR  latin* OR iraqi OR arab OR aboriginal* OR traveller* OR  gyps*  OR roma ) |
| 2 | **Population**  Children (aged 0-5) related | TITLE-ABS KEY (child OR "Infant" OR "Infant" OR "Minors" OR child* OR infan* OR young OR  paed* OR kid OR bab* OR pediat* OR preschool OR toddler* ) |
| 3 | **Intervention**  Intervention/Lifestyle modification related | TITLE-ABS-KEY ( "health Promotion"  OR  "social support"  OR  "family therapy"  OR  "primary prevention" OR "secondary prevention" OR "preventive measure*" OR prevent* OR "behav* chang*" OR "behave* interv*" OR "behav* program" OR "behavio*r chang*" OR "behavio*r interv*" OR "behavio*r program" OR "behav* modification" OR "behavio*r modification"  OR  "promotion of health" OR "promotional items" OR program* OR promot* OR "health promot*" OR "health Campaign*" OR campaign OR "soc* interv*" OR "lifestyle interv*" OR "lifestyle chang*" OR "lifestyle interv*" OR "life style chang*" OR "lifestyle intervention*" OR "behav* change techn*" OR "soc* support" OR "peer support" OR couns* OR "behav* couns*" OR "lifestyle couns*" OR "couns* educ*" OR "diet advice" OR "diet educ*" OR "health educ*" OR "behav* educ*" OR "psychological interv*"  OR  "life style interv*" OR "lifestyle interv*" OR "lifestyle change" OR "life style change" OR "commun* intervention" OR "family interv*" OR "parent* interv*" OR "lifestyle mod*" OR "obesity prevent*" OR "weight manage*" OR "physical educat*" ) |
| 4 | Feeding/Physical Activity related | TITLE-ABS-KEY (exercis* OR  activ*  OR  "aerobic exercis*" OR "leisure activ*" OR "exercis* therapy" OR "physical fitness" OR "group exercis*" OR "physical activ*" OR "activity change" OR walk* OR run* OR  jog* OR danc* OR skip* OR swim* OR "outdoor activit*" OR "outdoor games" OR game* OR play* OR "play time" OR "play* games" OR "infant feed*" OR breastmilk OR breastfed OR breastfeed* OR breast-feed* OR breast-fed  OR  "human milk"  OR  "bottle fe*"  OR  "infant formula feeding" OR "cow* milk" OR fortify OR "health* diet" OR "wet nursing" OR "milk sharing"  OR  mealtime*  OR  feed*  OR  "feeding beh*"  OR  "feeding pattern*" OR "feeding practice*" OR  food* OR eat* OR diet* OR "eating beh*" OR "eating practic*" OR "diet* intake" OR "diet* habit*" OR "food habit*" OR "eating habit*" OR nutrit* OR "energy intake" OR "food intake" OR "macronutrient*" OR "micronutrient*" OR "health*nutrit*" OR "wean*" OR "complimentary feed*" OR "solid feed*" ) ) |
| 5 |  | #1 AND #2 AND #3 AND #4 |
| 6 |  | #1 AND #2 AND #3 AND #4  (LIMIT-TO ( EXACTKEYWORD ,  "Infant" )  OR  LIMIT-TO (EXACTKEYWORD , "Child, Preschool" ) OR LIMIT TO (EXACTKEYWORD , "Preschool Child" ) OR LIMIT-TO ( EXACTKEYWORD , "Infant, Newborn" ) OR LIMIT TO ( EXACTKEYWORD , "Newborn" ) )  AND ( LIMIT-TO ( PUBYEAR ,  2023-1995 ) |

#### Table 3: Search strategy CENTRAL <1995 to June Week 4 2023>

| **Number** | **Concepts** | **Key words and combinations** |
| --- | --- | --- |
| 1 | **Population**  Ethnicity related | MeSH terms: ethnicity [EXP], racial groups [EXP], ethnic and racial minorities [EXP], emigrants and immigrants [EXP], cultural diversity [EXP], refugees [EXP], African Americans [EXP], Arabs [EXP], Asian Americans [EXP], Indigenous peoples [EXP], Jews [EXP], Roma [EXP], Blacks [EXP], American Native Continental Ancestry Groups [EXP], Asians [EXP], Native Hawaiian or Other Pacific Islander [EXP], racial groups [EXP], |
|  |  | Free text terms (ab.ti.kw): ethnic*, ethnic* group, minorit*, “ethnic* minorit*, minorit* group, rac* minorit*, foreign*, cultur*, diversit* B*ME, black Asian Minority Ethnic, multiethnic*, multi* ethnic*, mixed ethnic*, multi rac*, multi*cultural, multicultural, cross cultural, transcultural, emigra*, immigra*, migra*, asylum seeker, indigenous, refugee, transient, black, Afr*, Caribbean, Asian, Pakistani, Bangladesh*, India*, Chin*, Sri Lanka*, South Asia, Hispanic, Latin*, Iraqi, Arab, Aboriginal*, Traveller*, Gyps*, Roma |
| 2 | **Population**  Children (aged 0-5) related | MeSH terms: child [EXP], infant [EXP], infant [EXP], nurseries, minors |
|  |  | Free text terms (ab.ti.): child*, infan*, young, paed*, kid, bab*, pediat*, preschool, toddler* |
| 3 | **Intervention**  Intervention/Lifestyle modification related | MeSH terms: health promotion [EXP], social support [EXP], family therapy [EXP] |
|  |  | Free text terms (ab.ti.kw): primary prevention, secondary prevention, preventative measure*, prevent*, behav* chang*, behave* intervention, behav* program, behavio*r chang*, behavio*r interv*, behavio*r program, “behav* modification, behavio*r modification, promotion of health, promotional items, program*, promot*, health promot*, health Campaign*, campaign, soc* interv*, lifestyle interv*, lifestyle chang*, lifestyle intervention, life style chang*, behav* change techn*, soc* support, peer support, couns*, behav* couns*, lifestyle couns*, couns* educ*, diet advice, diet educ*, health educ*, behav* educ*, psych* interv*, lifestyle change, life style change, commun* interv*, family interv*, parent* interv*, lifestyle mod*, obesity prevent*, physical educat* |
| 4 | Feeding/Physical Activity related | MeSH terms: exercise [EXP], breast Feeding [EXP], bottle feeding, food preferences, ‘diet, healthy’, food fussiness |
|  |  | Free text terms (ab.ti.kw.): exercis*, activ*, aerobic exercis*, leisure activ*, exercis* therapy, physical Fitness, group exercis*, physical activ*, activity change, walk*, run*, jog*, danc*, skip*, swim*, outdoor activit*, outdoor game*, game*, play*, play time, play* games, breastmilk, breastfed, breastfeed*, breast-feed*, breast-fed, Infant feed*, human milk, bottle fe*, infant formula feeding, “cow* milk, fortify, wet nursing, milk sharing, mealtime*, feed*, feeding beh*, feeding pattern*, feeding practice*, food*, eat*, diet*, eating beh*, eating practic*, diet* intake, diet* habit*, food habit*, eating habit*, nutrit*, energy intake, food intake, macronutrient*, micronutrient*, health* nutrit*, wean*, complim* feed*, solid feed* |
| 5 |  | #1 AND #2 AND #3 AND #4 |
| 6 |  | #1 AND #2 AND #3 AND #4 with Publication Year from 1995 to present, with Cochrane Library publication date Between Jan 1995 and July 2023, in Trials |

NB: Search terms were combined with ‘OR’ and concepts were combined with ‘AND’

#### Table 4: Search strategy Medline <1995 to June Week 4 2023>

| **Number** | **Concepts** | **Key words and combinations** |
| --- | --- | --- |
| 1 | **Population**  Ethnicity related | MeSH terms: ethnicity [EXP], racial groups [EXP], ethnic and racial minorities [EXP], emigrants and immigrants [EXP], cultural diversity [EXP], refugees [EXP], African Americans [EXP], Arabs [EXP], Asian Americans [EXP], Indigenous peoples [EXP], Jews [EXP], Roma [EXP], Blacks [EXP], American Native Continental Ancestry Groups [EXP], Asians [EXP], Native Hawaiian or Other Pacific Islander [EXP], racial groups [EXP], |
|  |  | Free text terms: (("ethnic*"[Title/Abstract] OR "ethnicity"[Title/Abstract] OR "ethnic Group"[Title/Abstract]) OR ("minorit*"[All Fields] OR (("ethnical"[All Fields] OR "ethnically"[All Fields] OR "ethnicities"[All Fields] OR "ethnicity"[MeSH Terms] OR "ethnicity"[All Fields] OR "Ethnic"[All Fields] OR "ethnics"[All Fields] OR "ethnology"[MeSH Subheading] OR "ethnology"[All Fields] OR "ethnology"[MeSH Terms]) AND "minorit*"[All Fields]) OR ("minorit*"[All Fields] AND ("group s"[All Fields] OR "grouped"[All Fields] OR "grouping"[All Fields] OR "groupings"[All Fields] OR "groups"[All Fields] OR "groups s"[All Fields] OR "population groups"[MeSH Terms] OR ("population"[All Fields] AND "groups"[All Fields]) OR "population groups"[All Fields] OR "group"[All Fields])) OR "racial minorit*"[All Fields] OR ("foreign*"[All Fields] AND "rac"[All Fields]) OR "racial*"[All Fields]) OR ("cultur*"[Title/Abstract] OR "diversit*"[Title/Abstract] OR "BAME"[Title/Abstract] OR (("blackness"[All Fields] OR "blacks"[All Fields] OR "black"[All Fields]) AND "asian minority ethnic"[Title/Abstract])) OR ("multiethnic"[Title/Abstract] OR "multi ethnic*"[Title/Abstract] OR "mixed ethnic*"[Title/Abstract] OR "multi rac*"[Title/Abstract] OR ("multi*"[All Fields] AND "cultural"[Title/Abstract]) OR "multi cultural"[Title/Abstract] OR ("cross*"[All Fields] AND "cultural"[Title/Abstract]) OR "cross cultural"[Title/Abstract] OR "trans cultural*"[Title/Abstract] OR "transcultural"[Title/Abstract]) OR ("emigra*"[Title/Abstract] OR "immigra*"[Title/Abstract] OR "migra*"[Title/Abstract] OR "Asylum Seeker"[Title/Abstract]) OR ("indigenous"[Title/Abstract] OR "refugee"[Title/Abstract]) OR "transient"[Title/Abstract] OR ("black"[Title/Abstract] OR "afri*"[Title/Abstract] OR "Caribbean"[Title/Abstract]) OR ("Asian"[Title/Abstract] OR "pakistani"[Title/Abstract] OR "bangladesh*"[Title/Abstract] OR "india*"[Title/Abstract] OR "china"[Title/Abstract] OR "chinese"[Title/Abstract] OR "sri lanka*"[Title/Abstract] OR "south Asia"[Title/Abstract]) OR ("hispanic"[Title/Abstract] OR "latin*"[Title/Abstract]) OR ("Iraqi"[Title/Abstract] OR "arab"[Title/Abstract]) OR "aboriginal*"[Title/Abstract] OR ("traveller*"[Title/Abstract] OR "gyps*"[Title/Abstract] OR "roma"[Title/Abstract])) |
| 2 | **Population**  Children (aged 0-5) related | MeSH terms: child [EXP], infant [EXP], infant [EXP], nurseries, minors |
|  |  | Free text terms: ("child*"[Title/Abstract] OR "infan*"[Title/Abstract] OR "young"[Title/Abstract] OR "paed*"[Title/Abstract] OR "kid"[Title/Abstract] OR "pediat*"[Title/Abstract] OR "toddler*"[Title/Abstract] OR "baby*"[Title/Abstract] OR "babi*"[Title/Abstract])) |
| 3 | **Intervention**  Intervention/Lifestyle modification related | MeSH terms: health promotion [EXP], social support [EXP], family therapy [EXP] |
|  |  | Free text terms: ("primary prevention"[Title/Abstract] OR "secondary prevention"[Title/Abstract]) OR ("preventive measure*"[Title/Abstract] OR "preventative measure*"[Title/Abstract]) OR "prevent*"[Title/Abstract] OR ("behav*"[Title/Abstract] OR "behaviour"[Title/Abstract] OR "chang*"[Title/Abstract] OR "intervention*"[Title/Abstract] OR "program*"[Title/Abstract] OR "modification"[Title/Abstract]) OR "promotion of Health"[Title/Abstract] OR "promotional Items"[Title/Abstract] OR "program*"[Title/Abstract] OR "promot*"[Title/Abstract] OR "health promot*"[Title/Abstract] OR "health campaign*"[Title/Abstract] OR "campaign"[Title/Abstract] OR ("promotion of Health"[Title/Abstract] OR "promotional Items"[Title/Abstract]) OR ("soci*"[All Fields] AND "interv*"[Title/Abstract]) OR (("lifestyle interv*"[All Fields] AND "or"[All Fields] AND "lifestyle"[Title/Abstract]) OR "life style"[Title/Abstract] OR "chang*"[Title/Abstract] OR "intervention*"[Title/Abstract]) OR ("behav*"[All Fields] AND "change techn*"[Title/Abstract]) OR ("soci*"[All Fields] AND "support"[Title/Abstract]) OR "peer support"[Title/Abstract] OR "couns*"[Title/Abstract] OR ("behav*"[All Fields] AND "couns*"[Title/Abstract]) OR "lifestyle couns*"[Title/Abstract] OR ("couns*"[All Fields] AND "educ*"[Title/Abstract]) OR "diet advice"[Title/Abstract] OR "diet educ*"[Title/Abstract] OR "health educ*"[Title/Abstract] OR ("behav*"[All Fields] AND "educ*"[Title/Abstract]) OR "psychological interv*"[Title/Abstract] OR ("life style interv*"[All Fields] OR "lifestyle interv*"[All Fields] OR "lifestyle change"[All Fields] OR "life style change"[Title/Abstract]) OR ("commun*"[All Fields] OR “intervention" [Title/Abstract]) OR "family interv*"[Title/Abstract] OR "parent interv*"[Title/Abstract] OR "lifestyle mod*"[Title/Abstract] OR "obesity prevent*"[Title/Abstract] OR "weight manage*"[Title/Abstract] OR "physical educat*"[Title/Abstract]) |
| 4 | Feeding/Physical Activity related | MeSH terms: exercise [EXP], breast Feeding [EXP], bottle feeding, food preferences, ‘diet, healthy’, food fussiness |
|  |  | Free text terms  ("exercise"[MeSH Major Topic] OR "exercis*"[Title/Abstract] OR "activ*"[Title/Abstract] OR "leisure activ*"[Title/Abstract] OR "physical Fitness"[Title/Abstract] OR "group exercis*"[Title/Abstract] OR "physical activ*"[Title/Abstract] OR "activity change"[Title/Abstract] OR "walk*"[Title/Abstract] OR "run"[Title/Abstract] OR "jog"[Title/Abstract] OR "danc*"[Title/Abstract] OR "skip*"[Title/Abstract] OR "swim*"[Title/Abstract] OR (("outdoor"[Title/Abstract] "activit*"[Title/Abstract]) OR "games"[Title/Abstract]) OR "game*"[Title/Abstract] OR "play*"[Title/Abstract] OR ("play*"[All Fields] AND "and time"[Title/Abstract]) OR ("play*"[All Fields] AND "and games"[Title/Abstract]) OR "infant feed*"[Title/Abstract] OR "wet nursing"[Title/Abstract] OR "milk sharing"[Title/Abstract] OR "mealtime*"[Title/Abstract] OR ("feed*"[Title/Abstract] OR "feeding beh*"[Title/Abstract] OR "feeding pattern*"[Title/Abstract] OR "feeding practice*"[Title/Abstract]) OR ("food*"[All Fields] OR "eat"[All Fields] OR "diet*"[Title/Abstract]) OR ("eating beh*"[Title/Abstract] OR "eating practic*"[Title/Abstract]) OR (("nutrit*"[Title/Abstract] OR "energy intake"[Title/Abstract] OR "food intake"[Title/Abstract] OR "macronutrient*"[Title/Abstract] OR "micronutrient*"[All Fields]) AND "health nutrit*"[Title/Abstract]) OR ("wean*"[Title/Abstract] OR "complimentary feed*"[Title/Abstract] OR "solid feed*"[Title/Abstract]) OR (("diet*"[All Fields] AND "intake"[Title/Abstract]) OR "diet habit*"[Title/Abstract] OR "food habit*"[Title/Abstract] OR "eating habit*"[Title/Abstract]) OR ("bottle feed*"[Title/Abstract] OR "infant formula feeding"[Title/Abstract] OR "cow milk"[Title/Abstract] OR "fortify"[Title/Abstract]) OR "walk*"[Title/Abstract] OR ("exercis*"[All Fields] AND "therapy"[Title/Abstract]))) |
| 5 |  | #1 AND #2 AND #3 AND #4 |
| 6 |  | #1 AND #2 AND #3 AND #4 AND ((newborn[Filter] OR allinfant[Filter] OR infant[Filter] OR preschoolchild[Filter]) AND (2022:2023[pdat])) |
