# Supplementary material for: Effects of behavioural interventions for preventing obesity in young children from ethnic minority backgrounds: a systematic review of randomised controlled trials
Source: Arch Public Health. 2026 May 18;84:153. doi: 10.1186/s13690-026-01951-x (PMC13352745; doi:10.1186/s13690-026-01951-x)
Supplement: Supplementary file 3 — Supplementary Material 3. [file 13690_2026_1951_MOESM3_ESM.docx]

**Supplementary File 2: Risk of Bias (CASP)**

|  | **Author, Year** | **Section A**  *Is the study design valid for an RCT?* | | | **Section B**  *Was the methodology sound?* | | | **Section C**  *What are the results?* | | | **Section D**  *Will the results help locally?* | | **Overall Quality**  High (>80%); Moderate (50-79%); Low (<50%) | **Appraisal comments** |
| --- | --- | --- | --- | --- | --- | --- | --- | --- | --- | --- | --- | --- | --- | --- |
|  |  | **Q1** | **Q2** | **Q3** | **Q1** | **Q2** | **Q3** | **Q1** | **Q2** | **Q3** | **Q1** | **Q2** |  |  |
| 1 | Toussaint et al. 2021^1^ | ✓ | ✓ | ✓ | ✓ | ✓ | ✓ | ✓ | ✓ | X | ✓ | X | High | Well-designed study, although limited impact was shown in relation to BMI and food-related practices. |
| 2 a/b/c | Messito et al. 2020^2,3^; Gross et al. 2016^4^ | **✓** | **✓** | **✓** | **?** | **✓** | **✓** | **✓** | **✓** | **✓** | **✓** | **?** | High | Limited details on blinding. There was a positive impact on BMI, feeding practices, knowledge and feeding styles although BMI was not sustained. |
| 3 | Natale et al. 2014^5^ | ✓ | ? | ✓ | ? | X | ✓ | ✓ | ✓ | ? | ✓ | ? | Moderate | The between-group difference at baseline (age, obesity) could create a potential bias; no power calculation was conducted. Some positive results were found. |
| 4 | Vaughn et al. 2021^6^ | ✓ | ✓ | ✓ | ✓ | X | ✓ | ✓ | ✓ | X | ✓ | X | Moderate | The between-group difference at baseline (ethnicity, income, parental marital status) could create a potential bias; Limited benefits shown. |
| 5 | Tomayko et al. 2016^7^ | ✓ | ✓ | ✓ | ✓ | ✓ | ✓ | ✓ | X | ✓ | ✓ | ✓ | High | The precision of the estimate of the  intervention effect reported not reported; positive impact in relation to BMI which was sustained |
| 6 | Wasser et al. 2020^8^ | **✓** | **✓** | **✓** | **X** | **✓** | **✓** | **✓** | **✓** | **X** | **✓** | **X** | Moderate | Well-designed study overall. Investigators/data collectors were not blinded. Limited intervention effect noted. |
| 7a/b/c | Annesi et al. 2013^9-11^ | **✓** | **?** | **?** | **?** | **✓** | **?** | **X** | **X** | **✓** | **✓** | **✓** | Low | Unclear how if all participants were accounted for at conclusion. No power calculation. Control group was variable. |
| 8 | Barkin et al. 2012^12^ | **✓** | **✓** | **✓** | **X** | **✓** | **✓** | **✓** | **✓** | **✓** | **✓** | **✓** | Moderate | Investigators/data collectors not blinded. Underpowered to test changes in weight categories over the 3 months. |
| 9 a/b | Barkin et al. 2018^13^ Heerman et al. 2020^14^ | **✓** | **✓** | **✓** | **✓** | **✓** | **✓** | **✓** | **✓** | **X** | **✓** | **?** | High | Some positive impact for diet (recall) although no significant change in BMI found. |
| 10 | Beck et al. 2017^15^ | **✓** | **✓** | **?** | **✓** | **✓** | **✓** | **X** | **X** | **X** | **✓** | **?** | Moderate | The effect or precision of the estimate of the intervention not adequately reported. Some benefits noted. |
| 11 | Black et al. 2021^16^ | **✓** | **✓** | **✓** | **?** | **✓** | **✓** | **✓** | **✓** | **?** | **✓** | **?** | High | No details provided on blinding. Good benefits shown although not for BMI. |
| 12 | Bürgi et al. 2012^17^ | **✓** | **✓** | **✓** | **✓** | **✓** | **✓** | **✓** | **✓** | **✓** | **✓** | **✓** | High | Demonstrated good improvements although not for BMI. |
| 13 a/b | Davis et al. 2016^18^ Cruz et al. 2016^19^ | **✓** | **✓** | **✓** | **?** | **✓** | **✓** | **✓** | **✓** | **✓** | **✓** | **✓** | High | No details on blinding provided; positive results although only for self-reported outcomes |
| 14 | Fernandez-Jimenez et al. 2019^20^ | **✓** | **✓** | **✓** | **X** | **X** | **✓** | **✓** | **✓** | **✓** | **✓** | **✓** | Moderate | Investigators/data collectors not blinded; between-group difference at baseline (parental education, food security) could create a potential bias. |
| 15 | Fiks et al. 2017^21^ | **✓** | **✓** | **?** | **✓** | **✓** | **✓** | **✓** | **✓** | **✓** | **✓** | **✓** | High | Unclear if all participants who entered the study were accounted for at conclusion |
| 16 | Fisher et al. 2019^22^ | **✓** | **✓** | **?** | **✓** | **✓** | **✓** | **✓** | **✓** | **✓** | **✓** | **✓** | High | Unclear if all participants who entered the study were accounted for at conclusion; positive impact on calorie consumption. |
| 17a/b/c | Fitzgibbon et al. 2002; Stolley et al., 2003; Fitzgibbon et al., 2005^55-57^ | **✓** | **✓** | **✓** | **✓** | **✓** | **✓** | **✓** | **✓** | **✓** | **✓** | **✓** | High | Well-designed study; positive impact; intervention was effective in reducing BMI among overweight/obese children. |
| 18 a/b | Fitzgibbon et al. 2011^23^ Kong et al. 2016^24^ | **✓** | **✓** | **X** | **X** | **✓** | **✓** | **✓** | **✓** | **✓** | **✓** | **✓** | High | Only children with data at baseline and post intervention were included in primary analysis; participants/researchers not blinded to condition; no power calculation performed |
| 19 | Fitzgibbon et al. 2013^25^ | **✓** | **✓** | **X** | **X** | **✓** | **✓** | **?** | **✓** | **?** | **✓** | **X** | Moderate | Only children with data at baseline and post intervention were included in primary analysis; participants/researchers not blinded to condition; no power calculation performed |
| 20 | French et al. 2018^26^ | **✓** | **✓** | **✓** | **✓** | **✓** | **✓** | **✓** | **✓** | **✓** | **✓** | **✓** | High | Well-designed study; positive impact |
| 21 | Haines et al. 2016^27^ | **✓** | **✓** | **✓** | **X** | **✓** | **✓** | **✓** | **✓** | **✓** | **✓** | **✓** | Moderate | Not sufficiently powered; demonstrates positive impact |
| 22 | Heerman et al. 2019^28^ | **✓** | **✓** | **✓** | **✓** | **✓** | **✓** | **✓** | **✓** | **✓** | **✓** | **✓** | High | Well-designed study; demonstrates good impact for slower BMI growth and across all subjective outcomes. |
| 23 | Hughes et al. 2020^29^ | **✓** | **?** | **✓** | **?** | **✓** | **✓** | **✓** | **X** | **✓** | **✓** | **?** | Moderate | No details on randomisation or blinding; precision of the estimate of the intervention effect not reported; some positive changes noted (maternal feeding/ knowledge) |
| 24 | Esquivel et al. 2016^30^ | **✓** | **?** | **?** | **?** | **X** | **✓** | **X** | **X** | **X** | **✓** | **X** | Low | No details on blinding; the effect and precision of the estimate of the intervention effect were not reported; between-group difference at baseline (ethnicity) could create a potential bias; limited benefit noted. |
| 25 a/b/c | Natale et al. 2021; 2022^31^; 2017^32^ | **✓** | **✓** | **✓** | **✓** | **✓** | **✓** | **✓** | **✓** | **✓** | **✓** | **✓** | High | Well-designed study. Positive benefits noted although not for BMI trajectory. |
| 26 | Petrova et al. 2009^33^ | **✓** | **✓** | **?** | **?** | **X** | **X** | **✓** | **✓** | **X** | **✓** | **X** | Low | There were between-group difference at baseline (breastfeeding experience; intention to breastfeed) which could create a potential bias; did not achieve sample size for adequate power; limited impact. |
| 27 | Nix et al. 2021^34^ | **✓** | **✓** | **?** | **✓** | **✓** | **✓** | **✓** | **✓** | **✓** | **✓** | **✓** | High | Unclear if all participants who entered the study were accounted for at conclusion; positive results found. |
| 28 | Reifsnider et al. 2018^35^ | **✓** | **✓** | **?** | **X** | **✓** | **✓** | **✓** | **X** | **X** | **✓** | **X** | Moderate | Unclear if all participants who entered the study were accounted for at conclusion; no details on randomization provided; limited impact. |
| 29 | Rosenstock et al. 2021^36^ | **✓** | **✓** | **✓** | **X** | **✓** | **✓** | **✓** | **✓** | **✓** | **✓** | **✓** | High | Participants and study staff were not blinded to intervention; positive results reported. |
| 30 | Slusser et al. 2012^37^ | **✓** | **✓** | **✓** | **?** | **✓** | **✓** | **✓** | **X** | **✓** | **✓** | **✓** | High | No details on blinding; the precision of the estimate of the intervention effect was not reported; positive results although not sufficiently powered (pilot study). |
| 31 | Pallante et al. 2019^38^ | **✓** | **✓** | **✓** | **?** | **✓** | **✓** | **✓** | **X** | **X** | **✓** | **X** | Moderate | No details on blinding; the precision of the estimate of the intervention effect was not reported; limited benefit; not sufficiently powered (pilot study). |
| 32 | Colindres et al. 2011^39^ | **✓** | **✓** | **?** | **?** | **✓** | **✓** | **✓** | **x** | **✓** | **✓** | **✓** | Moderate | No details on blinding; the precision of the estimate of the intervention effect was not reported; positive results for both parents and children although not sufficiently powered (pilot study). |
| 33 | Alhassan et al. 2007^40^ | **✓** | **?** | **✓** | **?** | **✓** | **✓** | **✓** | **X** | **X** | **✓** | **X** | Moderate | Limited details on randomization and blinding; the precision of the estimate of the intervention effect was not reported; limited benefits noted although not sufficiently powered (pilot study). |
| 34 | Cepni et al. 2021^41^ | **✓** | **✓** | **✓** | **?** | **✓** | **✓** | **✓** | **✓** | **✓** | **✓** | **?** | High | Limited details on blinding; reveals positive results although not sufficiently powered (pilot study). |
| 35 | Harvey-Berino & Rourke, 2003^42^ | **✓** | **?** | **?** | **✓** | **✓** | **✓** | **✓** | **X** | **✓** | **✓** | **?** | Moderate | Lack of detail of how randomization was carried out; unclear if all participants who entered the study were accounted for at its conclusion; precision for treatment effect not reported; promising results although not sufficiently powered (pilot study). |
| 36 | Anderson et al. 2005^43^ | **✓** | **✓** | **✓** | **X** | **✓** | **✓** | **✓** | **✓** | **✓** | **✓** | **✓** | High | Investigators not blinded; no other details of blinding provided; positive impact on exclusive breastfeeding |
| 37 | Gago et al. 2023^44^ | **✓** | **✓** | **X** | **?** | **X** | **✓** | **✓** | **✓** | **X** | **✓** | **X** | Moderate | Study was halted early due to COVID-19. No details on blinding provided. Data was analysed as complete case analysis; however, there was limited missingness / no association found related to missingness. There were proportionately more families in the control who identified as Black, unemployed, and resided in a single-parent household. Limited effects noted, and only noted for those who received the highest dose. |
| 38 | Gans et al. 2022^45^ | **✓** | **✓** | **X** | **✓** | **✓** | **✓** | **✓** | **✓** | **✓** | **✓** | **✓** | High | There was no differential dropout between experimental groups and as such imputation of missing values was not included as part of the analyses. |

**NB: ✓– Yes; X – No; ? Can't tell**
