# Supplementary material for: Effects of behavioural interventions for preventing obesity in young children from ethnic minority backgrounds: a systematic review of randomised controlled trials
Source: Arch Public Health. 2026 May 18;84:153. doi: 10.1186/s13690-026-01951-x (PMC13352745; doi:10.1186/s13690-026-01951-x)
Supplement: Supplementary file 4 — Supplementary Material 4. [file 13690_2026_1951_MOESM4_ESM.docx]

# Supplementary file S4

##### Table 1 Behaviour change techniques mapped to all studies

| **Study**  **BCT** | **1** | **2** | **3** | **4** | **5** | **6** | **7** | **8** | **9** | **10** | **11** | **12** | **13** | **14** | **15** | **16** | **17** | **18** | **19** | **20** | **21** | **22** | **23** | **24** | **25** | **26** | **27** | **28** | **29** | **30** | **31** | **32** | **33** | **34** | **35** | **36** | **37** | **38** |
| --- | --- | --- | --- | --- | --- | --- | --- | --- | --- | --- | --- | --- | --- | --- | --- | --- | --- | --- | --- | --- | --- | --- | --- | --- | --- | --- | --- | --- | --- | --- | --- | --- | --- | --- | --- | --- | --- | --- |
| 1 Provide information on consequences of behaviour in general | +1 | 0 | +1 | +1 | 0 |  | +1 | +1 | 0 | +1 | 0 | +1 | +1 | +1 |  | +1 | 0 | 0 | 0 |  | 1 | +1 | +1 | +1 | +1 | +1 | +1 | +1 | +1 | 0 | +1 | +1 |  | 0 | +1 | 0 | +1 | 0 |
| 2 Provide information on consequences of behaviour to the individual |  | +1 |  |  |  |  |  |  |  |  |  |  |  |  |  |  |  |  |  |  |  | +1 |  |  | +1 |  | +1 | +1 | +1 | +1 |  |  |  |  |  |  |  |  |
| 3 Provide information about others approval |  |  |  |  |  |  |  |  |  |  |  |  |  |  |  |  |  |  |  |  |  |  |  |  |  |  |  |  |  |  |  |  |  |  |  |  |  |  |
| 4 Provide normative information about others behaviour |  |  |  |  |  |  |  |  |  |  | +1 |  |  |  |  |  |  |  |  |  |  |  |  |  |  |  |  |  |  |  |  |  |  |  |  |  |  |  |
| 5 Goal setting (behaviour) |  | +1 | +1 |  |  |  | +1 | +1 | +1 | +1 | 0 |  |  |  |  | +1 |  |  |  |  |  | +1 |  |  |  |  |  |  | +1 |  |  | +1 |  |  | +1 |  | +1 | 0 |
| 6 Goal Setting (outcome) |  |  |  |  |  |  |  |  |  | +1 | +1 |  |  |  |  |  |  |  |  |  |  |  |  |  |  |  |  |  |  |  |  |  |  |  |  |  |  |  |
| 7 Action Planning |  | +1 |  |  |  |  |  |  |  |  |  |  |  |  |  |  |  |  |  |  |  |  |  |  |  |  |  |  |  |  |  |  |  |  |  |  |  |  |
| 8 Barrier Identification/ Problem solving |  | +1 |  |  |  |  |  |  | +1 |  | +1 |  |  |  |  | +1 |  |  |  |  |  | +1 |  |  |  | +1 |  |  |  | +1 | +1 |  |  |  | +1 |  |  | 0 |
| 9 Set graded tasks |  |  |  |  |  |  | +1 |  |  |  |  |  |  |  |  |  |  |  |  |  |  |  |  |  |  |  | +1 |  |  |  |  | +1 |  |  |  |  |  |  |
| 10 Prompt review of behavioural goals |  |  |  |  |  |  |  | +1 |  |  | +1 |  |  |  |  | +1 |  |  |  |  |  |  |  |  |  |  |  |  |  |  |  |  |  |  |  |  |  | 0 |
| 11 Prompt review of outcome goals |  |  |  |  |  |  |  |  |  |  | +1 |  |  |  |  |  |  |  |  |  |  |  |  |  |  |  |  |  |  |  |  |  |  |  |  |  |  |  |
| 12 Prompt rewards contingent on effort or progress towards behaviour |  |  |  |  |  |  |  |  | +1 |  | 0 |  |  |  |  | +1 |  |  |  |  | +1 |  | +1 |  |  |  |  |  |  | +1 |  |  |  |  | +1 |  | +1 |  |
| 13 Provide rewards contingent on successful behaviour |  |  | +1 |  |  |  | +1 |  |  |  |  |  |  |  | +1 |  | +1 | +1 |  |  |  |  |  |  | 1 |  |  | +1 |  |  |  |  |  |  |  |  |  | 0 |
| 14 Shaping |  |  |  |  |  |  |  |  |  |  |  |  |  |  |  |  |  |  |  |  |  |  |  |  |  |  |  |  |  |  |  |  |  |  |  |  |  |  |
| 15 Prompt generalisation of a target behaviour |  |  |  |  |  |  |  |  |  |  |  |  |  |  |  |  |  |  |  |  |  |  |  |  |  |  |  |  |  |  |  |  |  |  |  |  |  |  |
| 16 Prompt self-monitoring of behaviour |  |  |  |  |  |  | +1 |  | +1 |  |  |  |  |  |  | +1 |  |  |  |  |  | +1 |  |  |  |  |  |  |  | +1 | +1 | +1 |  |  |  |  |  |  |
| 17 Prompt self-monitoring of behavioural outcome |  |  |  |  |  |  |  |  | +1 |  |  |  |  |  |  |  |  |  |  |  |  |  |  |  |  |  |  |  |  |  |  |  |  |  |  |  |  |  |
| 18 Prompt focus on past success | +1 | +1 |  |  |  |  |  |  |  |  |  |  |  |  |  |  |  |  |  |  |  |  |  |  |  |  |  |  |  |  |  |  |  |  |  |  |  |  |
| 19 Provide feedback on performance |  | +1 |  |  |  |  | +1 |  |  |  |  |  |  | +1 | +1 | +1 |  |  |  |  |  |  |  |  |  |  | +1 |  |  |  | +1 |  |  |  |  | +1 | +1 | 0 |
| 20 Provide information on where and when to perform behaviour | +1 |  |  |  |  |  |  |  |  | +1 |  |  |  |  |  |  |  |  |  |  |  |  |  |  |  |  |  |  |  |  |  |  |  |  |  |  |  |  |
| 21 Provide instruction on how to perform the behaviour | +1 | +1 |  |  | +1 |  |  |  |  |  | +1 |  | +1 |  |  |  |  |  |  |  |  |  |  | +1 | +1 | +1 | +1 | +1 |  | +1 | +1 |  |  | +1 |  |  |  | 0 |
| 22 Model/demonstrate behaviour |  | +1 | +1 |  |  |  |  | +1 |  | +1 | +1 |  | +1 |  | +1 | +1 | +1 | +1 | +1 |  |  |  |  |  |  |  |  |  |  | +1 | +1 |  |  | +1 | +1 | +1 |  | 0 |
| 23 Teach to use prompts/cues | +1 | +1 |  | +1 |  | 0 |  |  |  |  | +1 |  |  |  |  |  |  |  |  |  | +1 |  |  |  |  |  |  |  |  |  | +1 |  |  |  |  |  |  |  |
| 24 Environmental restructuring | +1 |  | +1 | +1 | 0 |  |  |  |  |  | 1 | +1 | +1 |  |  | +1 | +1 | +1 | +1 | +1 |  |  |  | +1 |  |  |  |  |  |  | 0 |  | +1 | +1 |  | +1 |  | 0 |
| 25 Agree to behavioural contract |  |  |  | +1 |  |  |  | +1 |  |  | 0 |  |  |  |  |  |  |  |  |  |  |  |  |  |  |  |  |  |  |  |  |  |  |  |  |  |  |  |
| 26 Prompt Practice | +1 | +1 | +1 | +1 |  |  |  | +1 |  |  | 0 | +1 |  |  | +1 |  | +1 | +1 | +1 |  |  |  |  |  |  |  |  | +1 |  |  |  |  |  |  |  |  | +1 | 0 |
| 27 Use of follow up prompts |  |  |  |  |  |  |  |  |  |  |  |  |  |  |  |  |  |  |  |  |  |  |  |  |  |  |  |  |  |  |  |  |  |  |  |  |  |  |
| 28 Facilitate social comparison |  |  |  |  |  |  |  |  |  |  | +1 |  |  | +1 | +1 | +1 |  |  |  |  |  |  |  |  |  |  |  |  |  |  |  |  |  |  |  |  |  |  |
| 29 Plan social support/ social change | +1 | +1 |  | +1 | 0 | 0 |  | +1 |  |  | +1 |  |  |  | +1 |  |  |  |  | +1 | +1 |  |  |  |  |  |  |  |  |  | +1 | +1 |  | +1 |  | 0 | +1 | 0 |
| 30 Prompt identification as a role model/ position advocate | +1 | +1 |  |  | +1 |  |  |  |  |  |  |  | +1 |  |  |  |  |  |  | +1 | +1 | +1 | +1 |  | +1 |  | +1 | +1 | +1 | +1 |  | +1 |  |  | +1 | 0 | +1 | 0 |
| 31 Prompt anticipated regret |  |  |  |  |  |  |  |  |  |  |  |  |  |  |  |  |  |  |  |  |  |  |  |  |  |  |  |  |  |  |  |  |  |  |  |  |  |  |
| 32 Fear arousal |  |  |  |  |  |  |  |  |  |  |  |  |  |  |  |  |  |  |  |  |  |  |  |  |  |  |  |  |  |  |  |  |  |  |  |  |  |  |
| 33 Prompt self-talk |  |  |  |  |  |  | +1 |  |  |  |  |  |  |  |  |  |  |  |  |  |  |  |  |  |  |  |  |  |  |  |  |  |  |  |  |  |  |  |
| 34 Prompt use of imagery |  |  |  |  |  |  |  |  |  |  |  |  |  |  |  |  |  |  |  |  |  |  |  |  |  |  |  |  |  |  |  |  |  |  |  |  |  |  |
| 35 Relapse prevention /coping planning |  |  |  |  |  |  |  |  |  |  |  |  |  |  |  |  |  |  |  |  |  |  |  |  |  |  |  |  |  |  |  |  |  |  |  |  |  |  |
| 36 Stress management / emotional control training |  |  |  |  |  |  | +1 |  |  |  |  |  |  |  |  | +1 |  |  |  |  |  |  |  |  |  |  |  |  |  |  |  | +1 |  |  |  |  |  |  |
| 37 Motivational interviewing |  | +1 |  |  |  |  |  |  | +1 |  |  |  |  |  |  |  |  |  |  | +1 |  |  |  |  |  |  |  |  |  |  |  |  |  |  |  |  |  | 0 |
| 38 Target management | +1 |  |  |  |  |  |  |  |  |  |  |  |  |  |  |  |  |  |  |  |  |  |  |  |  |  |  |  |  |  |  |  |  |  |  |  |  |  |
| 39 General communication skills training |  |  |  |  |  |  |  |  |  | +1 |  |  |  |  |  |  |  |  |  |  |  |  |  |  |  |  |  |  |  |  |  |  |  |  |  |  |  |  |

*NB: BCTs in experimental condition only +1; BCTs used in both the experimental and control condition 0; BCT used in the control group only 1^42^.*

##### Table 2: BCTs mapped to the Behaviour Change Wheel (BCW) using the Taxonomy Domains Framework (TDF)^44^

| Study  TDF | 1 | 2 | 3 | 4 | 5 | 6 | 7 | 8 | 9 | 10 | 11 | 12 | 13 | 14 | 15 | 16 | 17 | 18 | 19 | 20 | 21 | 22 | 23 | 24 | 25 | 26 | 27 | 28 | 29 | 30 | 31 | 32 | 33 | 34 | 35 | 36 | 37 | 38 |
| --- | --- | --- | --- | --- | --- | --- | --- | --- | --- | --- | --- | --- | --- | --- | --- | --- | --- | --- | --- | --- | --- | --- | --- | --- | --- | --- | --- | --- | --- | --- | --- | --- | --- | --- | --- | --- | --- | --- |
| Knowledge | ***✓*** | 🗙 | ***✓*** | ***✓*** | 🗙 | ***✓*** | ***✓*** | ***✓*** | 🗙 | ***✓*** | 🗙 | ***✓*** | ***✓*** | ***✓*** |  | ***✓*** | 🗙 | 🗙 | 🗙 |  | ⬤ | ***✓*** | ***✓*** | ***✓*** | ***✓*** | ***✓*** | ***✓*** | ***✓*** | ***✓*** | ***✓*** | 🗙 | ***✓*** |  | ***✓*** | ***✓*** | 🗙 | ***✓*** | 🗙 |
| Skills | ***✓*** | ***✓*** | ***✓*** | ***✓*** | 🗙 | ***✓*** | ***✓*** | ***✓*** | ***✓*** | ***✓*** |  |  | ***✓*** | ***✓*** | ***✓*** | ***✓*** | ***✓*** | ***✓*** | ***✓*** |  |  |  |  | ***✓*** |  |  | ***✓*** |  | ***✓*** | ***✓*** | ***✓*** | ***✓*** |  | ***✓*** | ***✓*** | 🗙 | ***✓*** | 🗙 |
| Memory, attention, and decision processes | ***✓*** | ***✓*** | ***✓*** |  |  |  |  |  |  |  |  |  |  |  |  |  |  |  |  |  |  |  |  |  |  |  |  |  |  |  |  |  |  |  |  |  |  |  |
| Behavioural regulation |  |  |  | ***✓*** | 🗙 | ***✓*** |  | ***✓*** | ***✓*** | ***✓*** | 🗙 | ***✓*** | ***✓*** | ***✓*** | ***✓*** | ***✓*** | ***✓*** | ***✓*** | ***✓*** |  |  |  |  |  |  |  |  |  |  | ***✓*** |  |  |  |  |  | ***✓*** | ***✓*** | 🗙 |
| Social influences | ***✓*** | ***✓*** |  | ***✓*** | 🗙 | 🗙 |  | ***✓*** |  |  | ***✓*** |  |  | ***✓*** | ***✓*** |  |  |  |  | ***✓*** | ***✓*** |  |  |  |  |  |  |  |  |  | ***✓*** | ***✓*** |  | ***✓*** |  | 🗙 | ***✓*** | 🗙 |
| Environmental Context and Resources |  |  |  | ***✓*** | 🗙 |  |  |  |  |  | 🗙 | ***✓*** | ***✓*** |  |  | ***✓*** | ***✓*** | ***✓*** | ***✓*** | ***✓*** |  |  |  | ***✓*** |  |  |  |  |  |  | 🗙 |  | ***✓*** |  |  | ***✓*** |  | 🗙 |
| Social/ Professional role and identity | ***✓*** | ***✓*** |  |  |  |  |  |  |  |  |  |  | ***✓*** | ***✓*** |  |  |  |  |  |  |  |  |  |  | ***✓*** |  | ***✓*** | ***✓*** | ***✓*** | ***✓*** |  | ***✓*** |  | ***✓*** |  |  |  | 🗙 |
| Beliefs about capabilities |  | ***✓*** |  |  |  |  | ***✓*** |  |  |  |  |  |  |  |  |  |  |  |  |  |  |  |  |  | ***✓*** |  |  |  |  |  |  |  |  |  |  |  |  | 🗙 |
| Optimism |  |  |  |  |  |  |  |  |  |  |  |  |  |  |  |  |  |  |  |  |  |  |  |  |  |  |  |  |  |  |  |  |  |  |  |  |  |  |
| Beliefs about consequences |  |  |  |  |  |  |  |  |  |  |  |  |  |  |  |  |  |  |  |  |  |  |  |  | ***✓*** |  |  |  |  |  |  |  |  |  |  |  |  |  |
| Intentions |  |  |  |  |  |  |  |  |  |  |  |  |  |  |  |  |  |  |  |  |  |  |  |  |  |  |  |  |  |  |  |  |  |  |  |  |  |  |
| Goals |  | ***✓*** |  | ***✓*** |  |  | ***✓*** | ***✓*** | ***✓*** | ***✓*** | 🗙 |  |  |  |  | ***✓*** |  |  |  |  |  | ***✓*** |  |  |  |  |  |  | ***✓*** |  |  | ***✓*** |  |  | ***✓*** |  | ***✓*** | 🗙 |
| Reinforcement | ***✓*** |  | ***✓*** |  |  |  | ***✓*** |  | ***✓*** |  | 🗙 |  |  |  | ***✓*** |  | ***✓*** | ***✓*** |  |  | ***✓*** | ***✓*** | ***✓*** |  | ⬤ |  |  | ***✓*** |  | ***✓*** |  |  |  |  | ***✓*** |  | ***✓*** | 🗙 |
| Emotion |  |  |  |  |  |  | ***✓*** |  |  |  |  |  |  |  |  |  |  |  |  |  |  |  |  |  |  |  |  |  |  |  |  |  |  |  |  |  |  |  |

# *N.B. ✓– Yes Intervention only; 🗙 in intervention and control; ⬤ in control only*
