# Supplementary material for: Effects of behavioural interventions for preventing obesity in young children from ethnic minority backgrounds: a systematic review of randomised controlled trials
Source: Arch Public Health. 2026 May 18;84:153. doi: 10.1186/s13690-026-01951-x (PMC13352745; doi:10.1186/s13690-026-01951-x)
Supplement: Supplementary file 5 — Supplementary Material 5. [file 13690_2026_1951_MOESM5_ESM.docx]

# Supplementary File 5

# *Figure 1: Percentage effectiveness ‘ratio’ of BCTs based on primary outcome*

# *n = number of studies that included each BCT domain*

## Figure 2 Percentage effectiveness ‘ratio’ of BCTs based on dietary intake

*n = number of studies that included each BCT domain*

## Figure 3: Percentage effectiveness ‘ratio’ of BCTs based on physical activity outcomes

*n = number of studies that included each BCT domain*

## Figure 4: Percentage effectiveness ‘ratio’ of BCTs based on infant feeding outcomes

*n = number of studies that included each BCT domain*
